# Genome and transcriptome delineation of two major oncogenic pathways governing invasive ductal breast cancer development

**DOI:** 10.18632/oncotarget.5543

**Published:** 2015-10-10

**Authors:** Luay Aswad, Surya Pavan Yenamandra, Ghim Siong Ow, Oleg Grinchuk, Anna V. Ivshina, Vladimir A. Kuznetsov

**Affiliations:** ^1^ Bioinformatics Institute (BII), Agency for Science, Technology and Research (A*STAR), Singapore 138671, Singapore; ^2^ School of Computer Engineering, Nanyang Technological University, Singapore 637553, Singapore

**Keywords:** invasive ductal carcinoma, low and high genetic grades, genome and transcriptome alterations, mutations, oncogenic pathway

## Abstract

Invasive ductal carcinoma (IDC) is a major histo-morphologic type of breast cancer. Histological grading (HG) of IDC is widely adopted by oncologists as a prognostic factor. However, HG evaluation is highly subjective with only 50%–85% inter-observer agreements. Specifically, the subjectivity in the assignment of the intermediate grade (histologic grade 2, HG2) breast cancers (comprising ~50% of IDC cases) results in uncertain disease outcome prediction and sub-optimal systemic therapy. Despite several attempts to identify the mechanisms underlying the HG classification, their molecular bases are poorly understood.

We performed integrative bioinformatics analysis of TCGA and several other cohorts (total 1246 patients). We identified a 22-gene tumor aggressiveness grading classifier (22g-TAG) that reflects global bifurcation in the IDC transcriptomes and reclassified patients with HG2 tumors into two genetically and clinically distinct subclasses: histological grade 1-like (HG1-like) and histological grade 3-like (HG3-like). The expression profiles and clinical outcomes of these subclasses were similar to the HG1 and HG3 tumors, respectively. We further reclassified IDC into low genetic grade (LGG = HG1+HG1-like) and high genetic grade (HGG = HG3-like+HG3) subclasses. For the HG1-like and HG3-like IDCs we found subclass-specific DNA alterations, somatic mutations, oncogenic pathways, cell cycle/mitosis and stem cell-like expression signatures that discriminate between these tumors. We found similar molecular patterns in the LGG and HGG tumor classes respectively.

Our results suggest the existence of two genetically-predefined IDC classes, LGG and HGG, driven by distinct oncogenic pathways. They provide novel prognostic and therapeutic biomarkers and could open unique opportunities for personalized systemic therapies of IDC patients.

## BACKGROUND

Invasive ductal carcinoma (IDC), the major histomorphologic type of breast cancer, is diagnosed in 180,000 women in the USA each year. The morphological assessment of the degree of tumor cell differentiation, represented by tumor histological grades (HGs), has attracted much attention for its potential to elucidate the heterogeneities of breast carcinoma (BC) due to its powerful prognostic capability, relative low cost, and simple methodology [1–5]. Moreover, HGs are considered to be effective for assessing tissue preference for metastasis and the genetic makeup of tumors [6–9]. Histological grading can be performed by combining cell morphology (nuclear polymorphism), tissue architecture (tubule formation) and visual assessment of the cell proliferation rate (mitotic count), but prognostic value of the combination of these features is still being discussed [1–3, 5, 10].

The HG of IDC is widely adopted by oncologists as a prognostic factor. However, HG evaluation is highly subjective with only 50%–85% inter-observer agreements [11]. The variability for the intermediate grade (histologic grade 2, HG2) breast cancers (comprising ~50% of IDC cases) is particularly evident, resulting in uncertain disease outcome prediction and sub-optimal systemic therapy.

In addition, HGs lack prognostic and predictive information for different intrinsic tumor subtypes classified as HG2 tumors, creating an uncertainty in cancer classification and prognosis [9, 10, 12–15]. On the other hand, numerous studies have shown significant associations between HG and patient survival as independent prognostic marker especially if the patients with HG1 and HG3 were compared [4, 6, 8, 16].

Several attempts have been made to identify the molecular mechanisms underlying the morphological characteristics of HG to improve its objectivity [1, 5, 6, 8, 17–19]. The continuous progressive model of tumor aggressiveness from low-grade to high-grade tumors has been accepted for the last few decades [20–22]. Alternatively, independent oncogenic pathways have been suggested based on observations of the differential loss of the 16q in HG1 versus HG3 tumors [1, 5, 18]. Previous genetic studies demonstrated the loss of 16q in HG1 IDC [1, 5, 17] and the possibility of micro-deletions in 16q in HG3 IDC [23]. For a detailed review of 16q loss frequency in different histological types of BC, the reader is referred to a review by Burger et al. [24]. However, there is still ambiguity regarding the intermediate HG2 tumors.

It was demonstrated that HG2 patients can be dichotomized based on gene expression profiles, with high accuracy (95%) into two genetically, and clinically distinct subclasses: histological grade 1-like (HG1-like) and histological grade 3-like (HG3-like) [6, 25, 26]. These subclasses, HG1-like and HG3-like, have similar gene expression profiles and clinical outcomes to HG1 and HG3 tumors, respectively. The 232 genes of grading classifier were involved mostly in cell cycle, p53 pathway, inhibition of apoptosis, cell adhesion, cell motility, stress, hormone response and angiogenesis [6, 25, 26].

Also, it has been argued that this genetic tumor aggressiveness grading classifier and its multiple representative 5–7 genes subsets can improve prognosis and therapeutic planning for BC patients diagnosed with tumor histologic type (HG2). Importantly, the patients have not been pre-selected based on any clinical characteristics (e.g., tumor stages, tumor size, ER and LN status). These re-classification results have been reproduced across different cohorts and treatment groups and strongly correlated with survival pattern of the re-classified tumor subgroups. Similar results were observed for the specific subpopulation of the BC selected by ER+ status [8, 27].

Collectively, genetic grade signatures can improve prognosis of BC patients, especially IDC patients with HG2 tumors, which are relatively poorly defined by different grading systems [6, 8, 26, 28].

Importantly, HG2 sub-classification studies supported the view that the low- and high-grade, defined via transcriptomic analysis, reflect independent patho-biological entities (distinct cell phenotypes) rather than a continuum of cancer progression [6, 25, 26].

Several studies have investigated the association of HG systems with DNA copy number variations (CNV) and mutation events [29, 30], but to our knowledge, no studies have reported a systematic interconnection of the CNV and mutation patterns in the HG2 of IDC.

To develop the concept of low- and high-grade tumors independence, we sought to provide a comprehensive transcriptome characterization of the low genetic grade (LGG, defined in this work as HG1 and HG1-like) and high genetic grade, HGG (defined in this work as HG3-like and HG3) tumors. This is an attempt to improve the objectivity of molecular grading of IDCs classes as well as narrowing the diagnostic, prognostic and predictive biomarkers spaces of IDC, specifying the differences between the tumors of each genetically-defined grade class. We also extend the characterization of these genetic classes to include stem-cell related genes, chromosome alterations and mutations that could differently drive their progression. Eventually, we discuss how these findings may boost our understanding of different cancer etiologies that lead to each genetic grade class and could help in the discovery of clinically-relevant biomarkers and improvement of current therapeutic strategies.

## RESULTS

### Feature selection methods and identification of the 22-gene tumor aggressiveness grading classifier

We studied the gene expression data of 430 TCGA IDC samples profiled using Agilent G4502A. The tumors consisted of the following histological grades: 32 HG1, 183 HG2 and 215 HG3 tumor samples (Table 1A).

**Table 1:** overview of the clinical information of TCGA cohort

A			
Parameter	Histological grade 1 (HG1)	Histological grade 2 (HG2)	Histological grade 3 (HG3)
**number of samples**	32	183	215
**ER+/ER−/NA**	31/0/1	163/17/3	127/85/3
**PGR+/PGR−/NA**	29/2/1	145/35/3	100/111/4
**HER2+/HER2−/ Her2(eq)/NA**	1/21/7/3	22/154/4/3	46/108/29/32
**Age median (SD)**	59 (13.2)	60 (13.8)	56 (12.6)
**stage I/II/III/IV/X/NA**	9/20/2/1/0/0	37/99/34/6/5/2	27/124/52/6/6/0
**LN+/LN−/NA**	11/21/0	93/90/0	115/99/1
**M+/M−/NA**	1/31/0	5/177/1	6/205/4

B			
Parameter	Genetic Grade 1-like (HG1-like)	Genetic Grade 3-like (HG3-like)	Genetic Grade 2 (GG2)
**number of samples**	101	78	4
**ER+/ER−/NA**	96/5/0	63/12/3	4/0/0
**PGR+/PGR−/NA**	89/12/0	53/22/3	3/1/0
**HER2+/HER2−/ Her2(eq)/NA**	6/92/2/1	15/59/2/2	1/3/0
**Age median (SD)**	61 (13.6)	58 (14)	53.5 (17)
**stage I/II/III/IV/X/NA**	25/52/18/2/2/2	12/46/14/4/2/0	0/1/2/0/1/0
**LN+/LN-**	53/48	37/41	3/1
**M+/M−/NA**	2/98/1	3/75/0	0/4/0

**A.** A summary of clinical parameters for the 430 IDC tumors of TCGA cohort for each histological grade. **B.** A summary of clinical parameters for the 183 IDC tumors of TCGA cohort for each subclass. **ER:** estrogen receptor. **PGR:** progesterone receptor. **HER2:** human epidermal growth factor receptor 2. **LN:** Lymph node. **M:** Metastasis. **NA:** not available. **eq:** equivocal. **SD:** standard deviation. ER, PgR and Her2 status were determined using Immunohistochemistry (IHC). Tumor stages determined according to American Joint Committee on Cancer system (AJCC). Histological grades were estimated using Nottingham Histologic Score, Scarff-Bloom-Richardson grading system (SBR), or Elston grading method.

**Figure 1:**
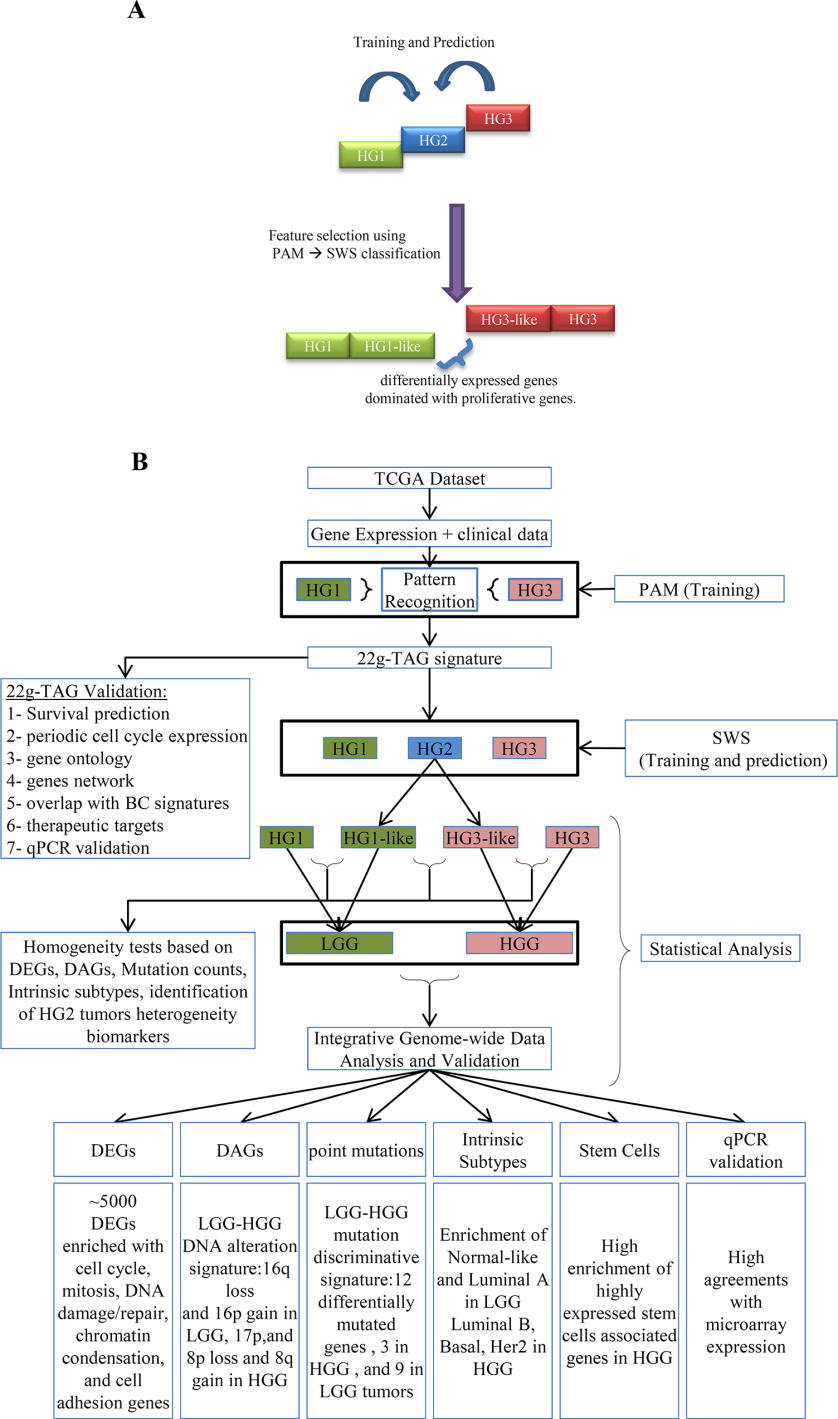
Schematic overview of the gene expression-based sub-classification of histological grade 2 (HG2) samples into HG1-like and HG3-like **A.** The basic concept of HG2 dichotomization based on the pattern recognition analysis supervised by the gene expression of HG1 and HG3 tumors. **B.** the workflow of our methodology of the sub-classification of HG2 and integrative data analyses of different genetic grades obtained by 22g-TAG classifier of TCGA cohort. DEGs: Differentially Expressed Genes; DAGs: Differentially Altered Genes; HG: Histological grades; SWS: Statistically Weighted Syndrome algorithm; PAM: Prediction Analysis of Microarray algorithm; LGG: Low Genetic Grades; HGG: High Genetic Grades.

In this study, we proposed that HG2 tumors are genetically heterogeneous and include tumors which oncogenic pathways could be separated into two distinct subclasses similar to either HG1 or HG3 tumors. To test this hypothesis for TCGA dataset, we applied a trained pattern recognition classifier to the intermediate HG2 tumors and evaluate the ability of the classifier to stratify HG2 tumors into HG1-like or HG3-like tumors (Figure 1A).

The workflow of our analysis is presented in Figure 1B. Due to the high dimensionality of the feature space (*n* = 90,797 probesets), we used a two-step analysis consisting of 1) feature selection procedure to reduce the biomarker space and 2) pattern recognition analysis for training a classifier to distinguish between two tumor classes. The number of patients with HG1 tumors was much smaller (32 patients) than in HG3 tumors (215 patients), demonstrating the imbalanced training set. It is known, that balanced dataset is very important for creating a robust and accurate training set [31]. To overcome the imbalance in the classes size of the training data, under-sampling of the majority classes were performed to avoid the bias in training accuracy toward the majority class [31].

Addressing the imbalance problem [31, 32], our method shuffled the 215 HG3 tumor expression profiles and separated them into seven non-overlapping (independent) subgroups (Supplementary Figure S1). First, our method used the prediction analysis of microarray (PAM) [33, 34]. The algorithm selects the most differentially expressed genes (DEG) (represented by the microarray probesets) that discriminate HG1 and HG3 tumors in our seven training sets. These training sets resulted in seven statistically reproducible classification signatures (The training accuracies and numbers of features are shown in Supplementary Table S1).

We selected 39 common probesets (corresponding to 22 genes) from the seven PAM-derived signatures. The 22 genes comprise *BUB1*, *CAPN8*, *CDC45*, *CDCA5*, *CDCA8*, *CENPA*, *CENPN*, *FAM72B/FAM72A*, *KIF13B*, *KIF14*, *KIF2C*, *MCM10*, *MELK*, *MTFR2*, *MYBL2*, *NAT1*, *NOSTRIN*, *ORC6*, *PIF1*, *SHCBP1*, *TICRR*,and *UBE2C*.

After reduction of the biomarker space to these 39 most representative probesets, we used the statistically weighted Syndrome (SWS) pattern recognition algorithm that outperforms PAM when a small number of features are used for training sets [6, 26]. It controls the stabilization of the prediction based on re-sampling and performs robustly in classification of small sample size of datasets [26, 35]. Similar to PAM analysis, SWS was performed for seven training/prediction sets to address the size imbalance of training classes. The average accuracy of SWS was 90.5 ± 3.4% (with average sensitivity of 90.2 ± 3.7%, average specificity of 91.5 ± 5.3%).

Next, during the prediction step, each HG2 tumor was assigned to either HG1-like or HG3-like sub-class. The overall prediction for each sample was based on the consensus agreement across the seven trained SWS classifiers. Consensus agreement is determined by the number of times a sample assigned to a given subclass with an assigning probability threshold (*p* ≥ 0.7). The tumor samples that showed predicted probability in an uncertainty zone (0.5 ± 0.2) was classified as “HG2-like” class. According to these criteria, 55.2% (101/183) and 42.6% (78/183) of HG2 tumors were assigned to HG1-like and HG3-like tumor type, respectively. The remaining 2.2% (4/183) of HG2 tumors could not be classified (interpreted as ‘true HG2′ and/or erroneous class). The distributions of the assigning probabilities of all training-prediction iterations are presented in Supplementary Figure S2. Summary of clinical information for HG2 tumors subclasses is shown in Table 1B.

**Figure 2:**
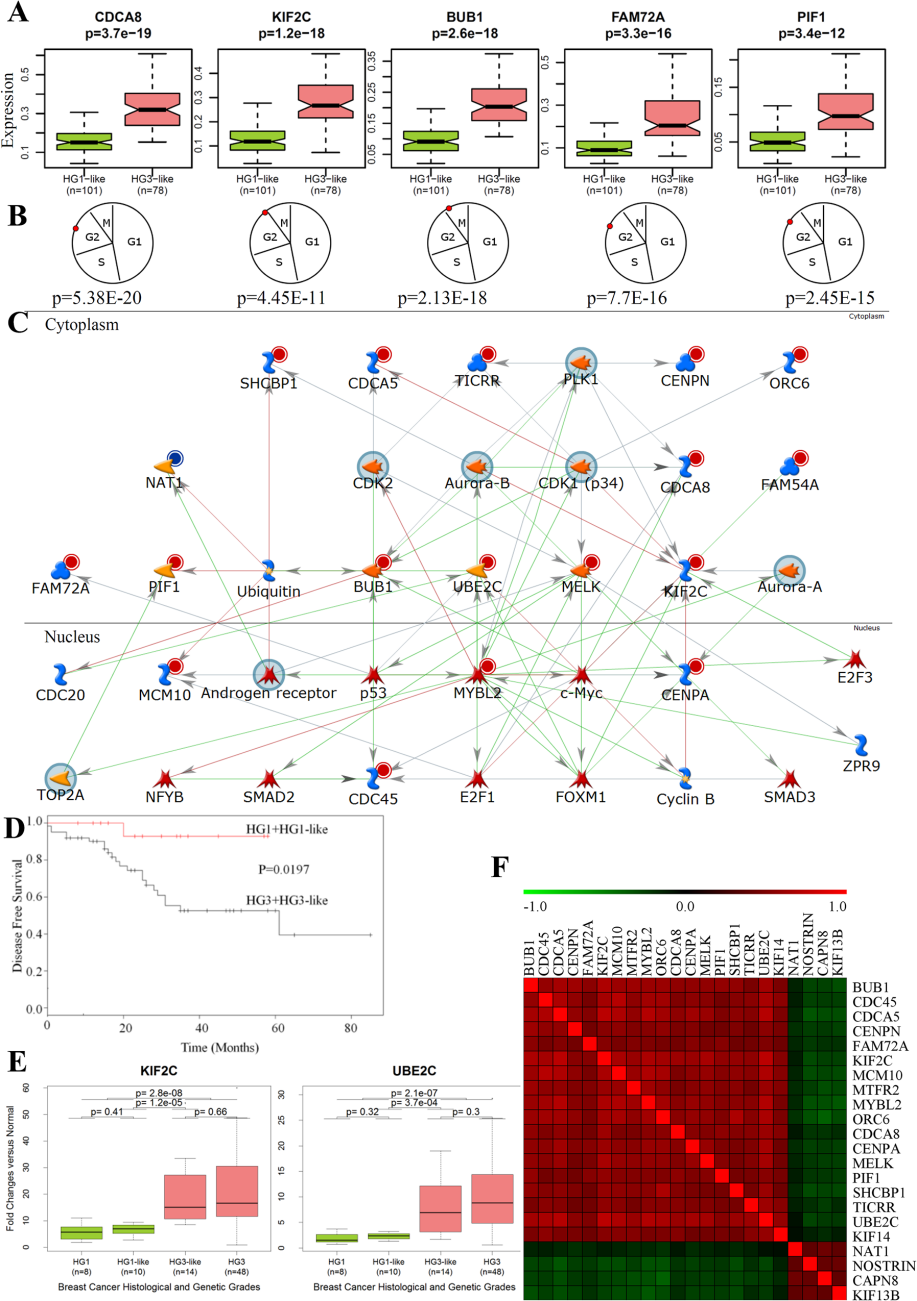
Functional and network analyses of 22g-TAG signature **A.** The differences in gene expression profiles between HG1-like and HG3-like samples for 5 genes from 22g-TAG signature. **B.** The peak expression of the 5 genes at the G2/M phase of the cell cycle. P is the *p*-value, which assesses the periodicity of a gene during the cell cycle according to the Cyclebase database. **C.** Network analysis of 22g-TAG signature genes using MetaCore network analysis tool. **D.** Kaplan-Meier curves of LGG and HGG patients' disease-free survival classified based on qPCR data of 22g-TAG genes **E.** Examples of the difference in qPCR-based expression for 2 genes of 22g-TAG for all histological and genetic grades of IDC patients. **F.** Heatmap of Kendall tau correlation coefficients between 22g-TAG genes using their qPCR-based relative expression profiles.

We provide a threshold of each probeset signal intensity value that signifies “low” or “high” expression level. The threshold expression values are important characteristics of medical classification system and they were listed in Supplementary Table S2A. We refer to this table as 22g-TAG classifier. All 22g-TAG genes are differentially expressed between HG1 and HG3 tumors (Supplementary Table S2A, examples of 5 genes are shown in Figure 2A)

### Comparison of the 22g-TAG classifier genes with 72 known signatures, including alternative molecular tumor grading signatures

To test the novelty of genes in 22g-TAG, we compared our 22g-TAG with reference lists of 72 BC gene signatures previously published in other studies and collated by our group [36, 37] (including 2 grading signatures from previous studies [18, 20]). Only one gene (*CAPN8*) can be considered a novel IDC-associated gene. Because most of 22g-TAG genes have been annotated as cell cycle genes, often considered the main hallmark of cancer, we assumed that a large proportion of 22g-TAG genes would be found in other gene signatures (Supplementary Table S3). Indeed, we found that *ORC6* (origin recognition complex, subunit 6 like (yeast)) and *PIF1* (5′-to-3′ DNA helicase homolog (*S*. *cerevisiae*) were observed in one of the 72 IDC signature gene lists. Consequently, they could also be considered “novel” BC-related genes and potential therapeutic targets. CAPN8 is a protease that plays a role in membrane trafficking of gastric cells and protection of gastric mucosa [38, 39]. PIF1 plays critical roles in DNA replication, cell growth, G-quadruplex, and R-loops resolving [40–42]. ORC6 is important cell cycle-related gene involves in DNA replication initiation and chromosome segregation [43, 44].

Interestingly, five genes (*CDC45*, *KIF13B*, *ORC6*, *SHCBP1*,and *CAPN8*) were not present in previously reported molecular tumor grading signatures [18, 20]. *MELK*, *MYBL2*, and *CDCA8* were the most common and were observed in 20, 18, and 16 BC signatures, respectively.

### 22g-TAG signature genes are potential prognostic markers

According to our data driven grouping (DDG) prognosis analysis (see Methods), all 22g-TAG genes were significant for patient survival (log-rank test FDR < 0.05) and showed consistent pattern (oncogene-like/tumor suppressor-like) in at least three of four independent validation cohorts (obtained from GEO dataset IDs: GSE1456 (Stockholm), GSE4922 (Singapore and Uppsala), and GSE21653 (Marseille)). Therefore, they could be considered as perspective prognostic markers (Supplementary Table S4). Moreover, the data-driven expression threshold values of survival prediction analysis of the genes and their mean expression in the low- and high-risk tumor development groups are significantly correlated (Kendal's tau correlation *p* < 0.05) among at least three cohorts (Supplementary Figure S3). Generally, the 22g-TAG signature outperformed other clinical parameters in the stratification of patients into prognostically meaningful groups, according to univariate and multivariate survival analyses based on Cox-regression model in at least three of the four validation cohorts (Supplementary Table S5). Collectively, the 22g-TAG signature genes are potentially reliable prognostic markers.

### 22g-TAG signature genes are involved in cell cycle/mitosis and oncogenic pathways

To study the biological relevance of the 22g-TAG genes, we performed gene ontology (GO) enrichment analysis and found that these genes are strongly enriched in cell cycle/mitosis gene ontology categories (*p* < 0.01, Supplementary Table S6).

Furthermore, using published datasets reporting the lists of periodically expressed cell cycle genes [45] and CycleBase database [46–48] containing experimentally defined cell cycle genes, we found that 80% (18/22) of 22g-TAG genes are periodically over-expressed in the cell cycle and show successive expression peaks within the cell cycle (mostly in the G2/M phase, Figure 2B, Supplementary Table S2B).

To further explore the relationships and interconnectivity among the signature genes and other cancer related genes, we conducted network analysis using the MetaCore software (Thomson Reuters, St. Joseph, MI). MetaCore includes manually curated knowledge database about annotated genes, their products and functional interactions. The 22g-TAG gene symbols were used as the seed nodes for “extension” of the gene network via finding the shortest path between any two genes of seed node set with maximum two intermediate nodes (genes or their products). Results showed strong association of 22g-TAG genes with key cancer-related genes such as *TP53*, *AURKA*, *TOP2A*, *E2F1*, and *MYC*, and that this network was generally associated with the mitotic cell cycle biological process (*p* = 9.1 × 10^−31^). *KIF2C* and *MYBL2* represent the convergence and divergence hubs, respectively for this network highlighting their role in IDC aggressiveness (Figure 2C). Two genes of 22g-TAG (*KIF2C* and *NAT1*) could be potentially druggable genes according to the drug-gene interaction database (DGIdb) [49], whereas 10 genes of 22g-TAG associated network are druggable (*AR, AURKA, AURKB, CDK1, CDK2, MYC, PLK1, SMAD2, TOP2A*, and *TP53*). Collectively, these analyses suggest that most 22g-TAG genes are molecularly interconnected and could act in concert with other genes during mitosis, specifically, during G2/M phases.

### Quantitative PCR-based validation of the 22g-TAG genes as a grading signature in an independent cohort

For further confirmation of the validity of the 22g-TAG signature as a tumor grading and prognostic signature, qPCR was conducted on 84 RNA samples of BC patients obtained from OriGene (see Methods). C_T_ values for each gene were obtained and normalized against endogenous control and normal tissue samples (*n* = 4) using the 2^−ΔΔCt^ method [50]. Obtained fold change values were used for the re-classification of HG2 samples using SWS algorithm. HG1 (*n* = 8) and HG3 (*n* = 48) tumors were used for training, and HG2 tumors (*n* = 24) were used as a class discovery set. Again, we used under-sampling to address the training classes' size imbalance. For that, HG3 samples were shuffled and split into 3 non-overlapping sets of 16 samples each. Three training-prediction sets were performed using SWS algorithm. HG2 tumors were finally sub-classified based on the consensus sub-classification of the three prediction iterations. The average training accuracy is 83.3% (sensitivity: 66.6 ± 7.2%, specificity: 91.7± 3.6%).

HG2 tumors (*n* = 24) were re-classified into HG1-like (*n* = 10) and HG3-like (*n* = 14) tumors. Because of the small number of HG2 samples, the prognostic survival levels of HG1-like and HG3-like patients were not significantly different. However, one of the HG1-like patients (10%) versus four of the HG3-like patients (28.6%) experienced a tumor relapse during the follow-up period. Furthermore, the survival difference between patients dichotomized onto low grade (HG1+HG1-like) and high grade (HG3-like+HG3) tumors is significant (log-rank test *p* = 1.9 × 10^−2^, Figure 2D).

Remarkably, all 22g-TAG genes show consistent expression trends across molecular grades according to both qPCR and microarray gene expression datasets (Supplementary Figure S4). Boxplots of the relative expression across different genetic grades for two genes are shown in Figure 2E. Expressions of all genes significantly correlate with each other based on qPCR data. Interestingly, oncogene-like genes correlate positively with each other but negatively with tumor suppressor-like genes, and vice versa (Figure 2F). Collectively, the sub-classification of HG2 into biologically and clinically meaningful classes by 22g-TAG signature genes is reproducible across different patients' cohorts and gene expression platforms.

Now, after we have assessed the validity of 22g-TAG as grading and prognostic signature, we will study the IDC/HG2 subclasses resulted from this signature.

### HG1-like and HG3-like tumors have distinct transcriptome profiles

We characterized HG1-like and HG3-like tumors, resulted from 22g-TAG, using integrative genomics and transcriptomics data analysis (Figure 1B). Starting with global gene expression profiles, we identified and studied differentially expressed genes (DEG) between HG1-like (*n* = 101) and HG3-like (*n* = 78) tumors. We selected 4,933 differentially expressed probesets based on the fold-changes (FC ≥ 1.25 or FC ≤ 0.75) and the statistical significance of two-tailed Wilcoxon test (Benjamini-Hochberg (FDR) < 0.01). These probeset signals correspond to RNA transcribed by 2147 genes: 887 genes (777 protein-coding, 26 pseudogenes, 33 ncRNA, 1 snoRNA, and 50 unknown transcripts) and 1,260 genes (1099 protein-coding, 83 pseudogenes, 18 ncRNA, and 60 unknown transcripts) were down-regulated and up-regulated, respectively, in HG3-like tumors with respect to HG1-like tumors (Supplementary Table S7).

GO enrichment analysis for the down-regulated genes revealed significant association with cell adhesion (Benjamini *p*-value = 5.5 × 10^−5^), extracellular matrix cellular component (Benjamini *p*-value = 4 × 10^−22^), focal adhesion pathway (Benjamini *p*-value = 8.5 × 10^−7^), cytoskeleton organization (Benjamini *p*-value = 7.7 × 10^−5^), and response to hormone stimulus (Benjamini *p*-value = 7.3 × 10^−4^). Up-regulated genes are strongly associated with the cell cycle (Benjamini *p*-value = 2.5 × 10^−66^), M phase (Benjamini *p*-value = 1.1 × 10^−56^), chromosome segregation (Benjamini *p*-value = 5.5 × 10^−23^), DNA repair biological processes (Benjamini *p*-values = 5.6 × 10^−16^), DNA replication pathway (Benjamini *p*-values < 1.2 × 10^−17^) and are related to the chromosome, kinetochore, and microtubule cellular components (Benjamini *p*-values < 1.1 × 10^−6^). Interestingly, the gene locations of up-regulated genes are enriched in specific chromosomes, such as chr8, chr17, chr20, and chr22 (Benjamini *p*-values < 3 × 10^−5^, Supplementary Table S8).

**Figure 3:**
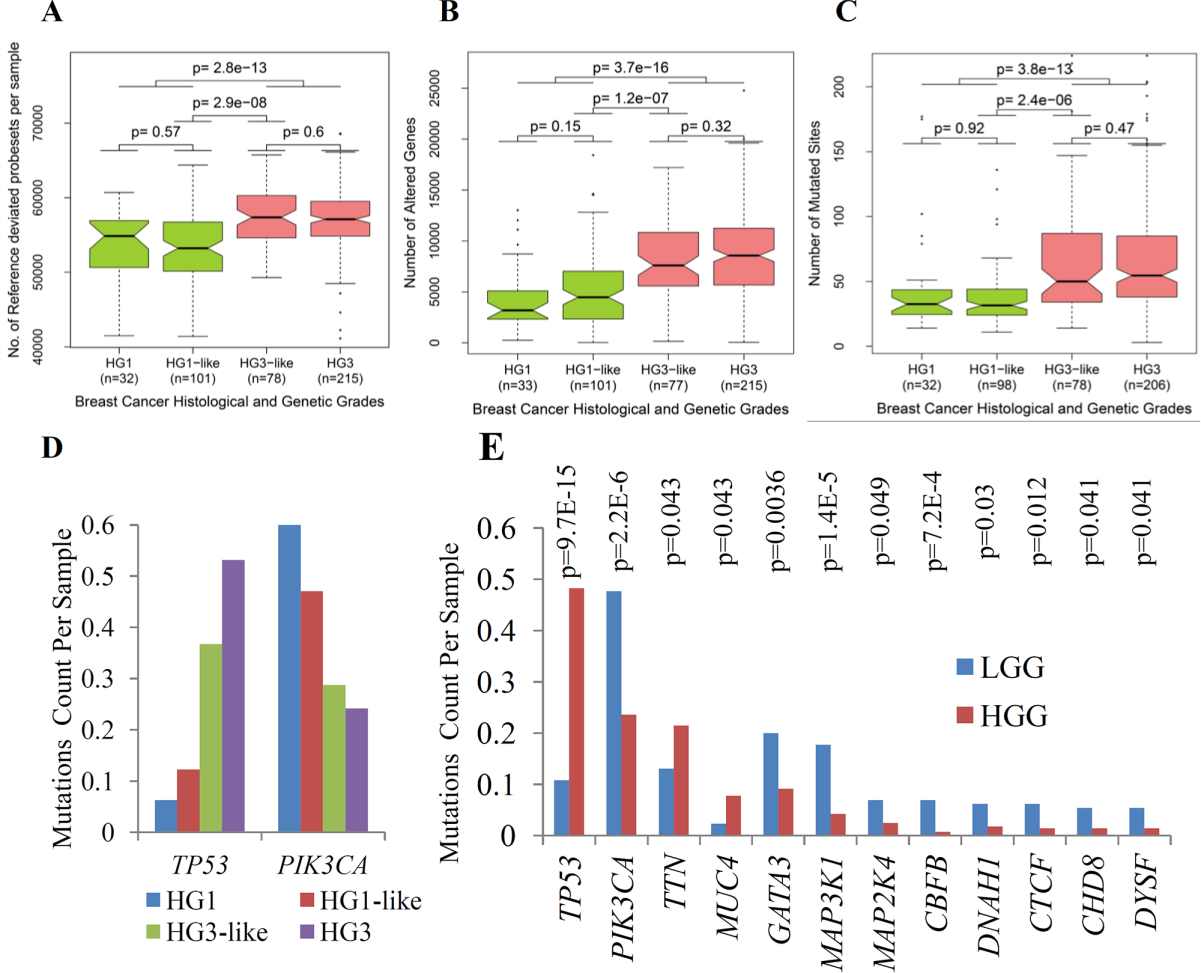
Major genomic and transcriptomic variations between subclasses of IDC determined by 22g-TAG classifier **A.** Box plots of the number of reference deviated genes (RDG) per sample for histological and genetic grades of IDC associated with 22g-TAG classifier. **B.** Box plots of the numbers of altered genes (AG) per sample for histological and genetic grades of IDC associated with 22g-TAG classifier. **C.** Box plots of mutations count per sample for histological and genetic grades of IDC associated with 22g-TAG classifier. The differences in the numbers of RDG, altered genes or mutations counts between different combinations of genetic grades were assessed statistically using two-tailed Wilcoxon test. **D.** Bar plots of mutations counts per sample for different genetic grades for *TP53* and *PIK3CA*. **E.** Bar plots of mutations counts per sample in LGG and HGG tumors for 12 genes that are correlated significantly with LGG and HGG classification. P is *p*-value of Fisher exact test.

We provide a quantitative measurement of the number of expressed genes per sample which expressions are deviated from reference (genes are represented by their assigned probesets). This reference is defined by the median values of the same genes in normal tissue to obtain a fold change profile with respect to the reference. Using fold change thresholds (FC ≥ 1.25 or FC ≤ 0.75) we count the number of genes that satisfied these criteria per sample. We call these genes as reference-deviated genes (RDG). We found that HG3-like tumors have significantly larger number of RDG than HG1-like tumors (*p* = 3 × 10^−8^, Figure 3A). Therefore, on the genome scale, HG1-like and HG3-like tumors have distinct gene expression profiles that were associated with distinct molecular functions when compared with each other and also with normal breast tissue.

### HG1-like and HG3-like tumors are distinct in their genomic constitution

We studied HG1-like and HG3-like tumors at the DNA level to characterize the DNA copy number variation (CNV) and point mutation events of each tumor subclass (Figure 1B). We studied Affymetrix human genome-wide SNP 6.0 array data for HG1-like (*n* = 101) and HG3-like (*n* = 77) tumors that were also profiled by gene expression. The CNV data of each individual sample were analyzed by Partek^®^ Genomics Suite^™^ software (see Methods). We transformed the CNV signal intensities into log2 values with respect to diploid status (i.e. transformed CNV signal intensity of diploid locus = 1). To determine whether a gene is amplified or deleted in a given tumor, thresholds of 1.25 for gene gain and 0.75 for gene loss were applied to CNV signal intensities of HG1-like and HG3-like tumors. As a primary analysis, to detect the differences between HG1-like and HG3-like tumors, the number of altered genes (gain or loss) in each sample was determined based on the previously mentioned thresholds. HG1-like tumors exhibited fewer altered genes (AG) per sample than HG3-like tumors. This difference in the overall number of AG was assessed statistically using two-tailed Wilcoxon test (*p* =1.2 × 10^−7^, Figure 3B). This significant difference was also observed in both the loss and gain of genomic regions (*p* = 9.5 × 10^−7^ and 7.8 × 10^−6^ for gene loss and gain, respectively; Supplementary Figure S5A).

Next, we studied 25,172 unique gene symbols (annotated genes resulting from segmentation analysis, see Methods) and identified individual genes that exhibit differential copy number status between HG1-like and HG3-like tumors. Differentially altered genes (DAG) were selected based on the following criteria: a) the median value of CNV intensity of either HG1-like or HG3-like tumors passes the thresholds for gain or loss (1.25 and 0.75, respectively), and b) the CNV profiles of HG1-like and HG3-like tumors are significantly different (*p* < 0.05). Our results revealed 1,214 DAG (925 protein-coding, 242 ncRNA, 32 pseudo, 14 snoRNA, and 1 snRNA, Supplementary Table S9). These genes include well-known altered genes important for BC initiation, development, and progression. For instance, the *TP53* gene, located on chromosome 17p, is deleted in 37% (37 of 101 samples) of HG1-like tumors and 64% (49 of 77 samples) of HG3-like tumors.

Further analysis of the genes' loci showed that many of these altered genes are enriched in a few chromosomes. Specifically in HG1-like tumors, there is a deletion of part of 16q. In contrast, HG3-like tumors showed gains in 8q, 17q, and 20q and losses in 8p, 11q, and 17p (Table 2A). It is notable that the chromosomes that harbor the DAG are the same chromosomes in which the DEG are enriched (chromosomes 8, 17, and 20).

**Table 2:** summary of differentially altered genes (DAG) of copy number variaiton

A) chromosome	Number of genes	chromosome bands
chr8	472	8p23.3, 8p23.1, 8p21.3–8p12, 8q13.1–8q24.3
chr11	334	11q21–11q25
chr17	333	17p13.3–17p11.2,17q23.1
chr16	45	16q12.1–16q13
chr20	20	20q13.13–20q13.2
chr1	4	1q21.1–1q21.3
**Total**	1208	–

B) chromosome	Number of genes	chromosome bands
chr16	11	distributed in q arm
chr1	1	–
**Total**	12	–

C) chromosome	Number of genes	chromosome bands
chr11	335	11q21–11q25
chr16	286	16q21–16q24.3
chr17	58	17p13.3–17p13.2
chr7	1	–
**Total**	680	–

D) chromosome	Number of genes	chromosome bands
chr1	1	–
chr16	971	16p13.3–16p11.2, 16q11.216q24.3
chr17	192	17p13.2–17p11.2
chr8	694	8p23.3,8p23.1–8p12,8q12.2–8q24.3
**Total**	1858	–

Summary of number of genes and their genomic location that showed differentially altered copy number profile across different genetic grades. **A.** the DAG between HG1-like and HG3-like tumors. **B.** the DAG between HG1-like and HG1 tumors. **C.** the DAG between HG3-like and HG3 tumors. **D.** the DAG between LGG and HGG tumors.

Therefore, genes within these chromosomes could be considered as the major players in initiation and maintenance of differential level of malignancy that distinguish between HG1-like and HG3-like tumors at both the DNA and mRNA levels.

### HG1-like and HG3-like tumors are distinct in their DNA point mutational profiles

Also, we conducted DNA point mutation analysis to study the mutations counts in HG1-like (*n* = 98) and HG3-like (*n* = 78) tumors (Figure 1B). We calculated the numbers of mutated sites (mutations counts) for all the genes in each tumor. Subsequently, we assessed the difference in the mutations counts in HG1-like and HG3-like tumors using two-tailed Wilcoxon test. We found that HG1-like tumors generally exhibited lower mutations counts than HG3-like tumors (*p* = 2.2 × 10^−6^, Figure 3C). This difference was consistent for the three most frequent types of mutations in our data-missense, nonsense, and silent mutations (*p* = 3.2 × 10^−6^, 6.4 × 10^−4^, and 1 × 10^−5^, respectively, Supplementary Figure S5B). The two most frequently mutated genes are *TP53* and *PIK3CA*. These two genes are the only genes that show a significant association in their mutational status with the sub-classification of HG2 tumors, as assessed using Fisher's exact test of independence. As we expected, *TP53* showed significantly lower mutations counts in HG1-like tumors (12 of 98 samples; 12.5%) than in HG3-like tumors (28 of 78 samples; 35.9%), (*p* = 1.5 × 10^−4^). However, *PIK3CA* exhibited higher mutations counts in HG1-like tumors (44 of 98 samples; 44.9%) than in HG3-like tumors (21 of 78 samples; 26.9%), (*p* = 1.4 × 10^−2^) (Figure 3D).

These results suggest essential differences in mutations frequency in *TP53* and *PIK3CA* provide mutagenesis background, strongly discriminating HG1-like from HG3-like tumors.

### Reclassified HG1-like and HG3-like tumors from the HG2 tumors are genetically comparable to HG1 and HG3 tumors, respectively

Figures 3A, 3B, 3C, and Supplementary Figure S5 show that there is no statistically significant differences between HG1 and HG1-like tumors as well as between HG3-like and HG3 with respect to RDG, AG, and mutation counts in all cases (*p*-values > 0.05). Moreover, no DEG between HG1 and HG1-like were detected whereas 1,837 DEG were detected between HG3-like and HG3 but these genes did not show significant enrichment in any biological process, cellular component, or pathway for the up-or down-regulated genes (Benjamini *p*-values > 0.01). Only a few molecular functions showed significant enrichment and are associated with ATP binding. Next, we performed unsupervised hierarchical clustering (HC) of expression profiles of all IDC presented in TCGA database. Only 4,933 probesets that were identified as differentially expressed expression signal in the comparison of HG1-like and HG3-like tumors were used for HC analysis. Using Euclidean distance and the average linkage agglomerative method, HC revealed two major clusters: 78% (104 of 133 samples) of HG1 and HG1-like tumors were enriched in one cluster, and 89% (261 of 293 samples) of HG3 and HG3-like tumors were enriched in the other cluster (Supplementary Figure S6). Large and positive value of Cohen's Kappa correlation coefficient suggests a high similarity between classification results of SWS and HC methods (*κ* = 0.67, *p* = 3.1 × 10^−43^; Supplementary Table S10). Thus, the results confirmed the 2-clusters pattern of all IDC derived due to 22g-TAG classifier.

We studied the DAG between HG1-like and HG1 tumors. Using the same criteria used for HG1-like and HG3-like tumors, we found only 12 significant DAG between HG1-like and HG1 tumors; 11 genes are on chromosome 16, and one is on chromosome 1 (Table 2B). Similarly, for HG3-like and HG3 tumors, we found 680 significant DAG enriched primarily in chromosomes 11, 16, and 17 (Table 2C). These results suggest more diversity between HG3-like and HG3 tumors than between HG1-like and HG1 tumors.

Generally, 16q loss occurred more frequently in HG1 and HG1-like tumors compared with HG3-like and HG3 tumors. For example, an important centromeric protein-encoding gene located on 16q, *CENPT*, exhibited loss in 88%, 70%, 54.5%, and 46.7% of HG1, HG1-like, HG3-like, and HG3 tumors, respectively (Fisher-exact test *p* = 1 × 10^−26^) and thus, the CNV of this gene locus could be used as structural biomarker of the aggressiveness of IDC.

Finally, we observed no correlation between HG1 and HG1-like according to the mutation status of all genes in the dataset. Similarly, for HG3-like and HG3 tumors, no genes showed any significant correlation between their mutation status and grade classification with the exception of *TP53* (*p*-value = 0.016). Thus, our results from the comparisons of HG1-like with HG1 tumors as well as HG3-like with HG3 tumors from the perspectives of transcript expressions, CNV or mutations revealed the relative homogeneity of HG1/HG1-like tumors and that of HG3/HG3-like tumors. Overall, these findings suggest multi-layered molecular dichotomization of IDC into LGG and HGG classes, predetermined by 22g-TAG classifier, specific patterns of DNA alterations and point mutations.

### Grading reclassification of IDC tumors correlates with intrinsic molecular subtypes and 16q loss

The intrinsic subtypes information of the tumors was obtained from TCGA network [51], of which PAM50 model [52] was used to achieve the classification for each sample. A contingency table of the frequency of 5 different subtypes (normal-like, luminal-A, luminal-B, basal-like, and HER2-enriched subtypes) versus the 4 classes of grading classification (HG1, HG1-like, HG3-like, and HG3) was generated. Luminal-A tumors are enriched and distributed in HG1 and HG1-like tumors (low genetic grade/LGG), whereas luminal-B, HER2-enriched, and basal-like tumors are enriched in HG3 and HG3-like tumors (high genetic grade/HGG). The association of LGG with luminal-A/normal-like and that of HGG with luminal-B/HER2-enriched/basal-like tumors is significant (Chi-square *p*-value = 4.3 × 10^−39^, Table 3A).

**Table 3:** the association of histological grades sub-classification and intrinsic subtypes

A) TCGA cohort	LGG		HGG	
Intrinsic Subtypes	HG1	HG1-like	HG3-like	HG3
**Normal-like**	0 (0%)	0 (0%)	0 (0%)	5 (2.8%)
**Luminal-A**	27 (87.1%)	92 (91.1%)	21 (26.9%)	33 (18.6%)
**Luminal-B**	4 (12.9%)	8 (7.9%)	32 (41%)	62 (35%)
**Basal-like**	0 (0%)	0 (0%)	8 (10.3%)	42 (23.7%)
**ERBB2+**	0 (0%)	1 (1%)	17 (21.8%)	35 (19.8%)
**Total**	31 (100%)	101 (100%)	78 (100%)	177 (100%)
			Chi-square test of homogeneity within HG3-like and HG3 tumors *p*-value = 0.06	
	Chi-square test of independence between LGG and HGG tumors versus Normal-like, Luminal A as one class and Luminal B, Basal-like, HER2-enriched tumors as another class *p*-value = 4.281E-39			

B) Uppsala and Stockholm cohorts	LGG		HGG	
Intrinsic Subtypes	HG1	HG1-like	HG3-like	HG3
**Normal-Like**	41 (37.3%)	43 (33.1%)	3 (5.6%)	1 (1%)
**No Subtype**	13 (11.8%)	23 (17.7%)	4 (7.4%)	1 (1%)
**Luminal-A**	43 (39.1%)	50 (38.5%)	12 (22.2%)	10 (9.9%)
**Luminal-B**	4 (3.6%)	6 (4.6%)	15 (27.8%)	27 (26.7%)
**ERBB2+**	6 (5.5%)	7 (5.4%)	9 (16.7%)	20 (19.8%)
**Basal-like**	3 (2.7%)	1 (0.8%)	11 (20.4%)	42 (41.6%)
**Total**	110 (100%)	130 (100%)	54 (100%)	101 (100%)
	Chi-square test of homogeneity within HG1-like and HG1 tumors *p*-value = 0.43		Chi-square test of homogeneity within HG3-like and HG3 tumors *p*-value = 0.25	
	Chi-square test of independence between LGG and HGG tumors versus Normal-like, Luminal-A as one class and Luminal-B, Basal-like, HER2-enriched tumors as another class *p*-value = 2.8e-45			

Breast cancer intrinsic subtype stratification according to histological and genetic grade classification of TCGA **A.** Uppsala, and Stockholm **B.** Breast cancer cohorts:Low-genetic grade LGG (HG1+HG1-like) and high-genetic grade HGG (HG3-like+HG3) are considered two major genetically distinct classes of BC.

In parallel, because of the small number of normal-like samples in TCGA cohort, we performed a similar analysis for the grading classification of the Uppsala and Stockholm cohorts studied previously by Ivshina et al., in which HG2 tumors were sub-classified based on their 5-genes grading signature [6]. Therefore, we compared the reclassified LGG and HGG tumors of the Uppsala and Stockholm cohorts with their intrinsic molecular subtypes (Table 3B). The results showed that 73.7% of LGG tumors (177 of 240 samples) were strongly associated with normal-like and luminal-A tumor subtypes. In contrast, 80% of HGG (124 of 155 samples) tumors are strongly associated with luminal-B, ERBB2+, and basal molecular tumor subtypes (Chi-square *p*-value = 2.8 × 10^−45^). The associations obtained from independent analyses of both TCGA and the Uppsala/Stockholm data are consistent (Table 3A and 3B).

To analyze the homogeneity of HG1 and HG1-like tumors with respect to intrinsic subtypes, we performed a chi-square test of homogeneity between HG1 and HG1-like subgroups and the enriched intrinsic molecular subtypes within them (*p* > 0.05, Table 3B). The lack of statistical significance suggests that HG1 or HG1-like subclasses could be similar. Similar results were observed for HG3 and HG3-like tumors (*p* > 0.05, Table 3A and 3B). Together, the homogeneity between HG1-like and HG1 tumors and the homogeneity between HG3-like and HG3 tumors seems to suggest the lack of a distinct intermediate grade between LGG and HGG. These results also provide plausible evidence to supporting the unlikelihood of inter-grade progression from the LGG to HGG classes.

Interestingly, this current subtype grouping was further corroborated by a study performed previously using a different classification method based on the expression of genes located on 16q [53], further suggesting that our sub-classification may also be associated with 16q copy number variation status.

### Gene expression, copy number variation, and mutation data provide a molecular basis for the genome wide re-classification of IDC into clinically distinct LGG and HGG tumor classes

We considered LGG and HGG tumor classes as the two major classes of IDC, which are supposed to have distinct genomic background and perhaps distinct oncogenic pathways, cellular functions, and therapeutic specificity. Therefore, we performed similar analyses of gene expression, CNV, and point mutations for these major classes (LGG *n* = 133 and HGG *n* = 293).

For DEG, we selected 14,357 (16% of 90,797 total probesets) differentially expressed probesets corresponding to 5,691 genes. Of these, 2,618 genes (2,285 protein-coding, 99 pseudogenes, 101 ncRNA, 2 snoRNA, and 131 unknown) and 3,073 genes (2,594 protein-coding, 187 pseudogenes, 84 ncRNA, 2 snoRNA, and 206 unknown) are down- and up-regulated, respectively, in HGG tumors with respect to LGG tumors (Supplementary Table S11).

GO functional enrichment analysis for down-regulated genes showed significant associations with cell adhesion (Benjamini *p*-value = 5.34 × 10^−11^), response to steroid hormone stimulus (Benjamini *p*-value = 3.2 × 10^−5^) biological processes, extracellular matrix and basement membrane cellular components (Benjamini *p*-value < 1.4 × 10^−3^), and with PDGF signaling pathway (Benjamini *p*-value = 3.9 × 10^−3^, Supplementary Table S12). Up-regulated genes are associated with cell cycle, chromosome segregation, and DNA replication biological processes (Benjamini *p*-value < 1.3 × 10^−14^) and involved in kinetochore and spindle microtubule cellular components (Benjamini *p*-value < 1.9 × 10^−3^), and the genes are strongly enriched among the genes expressed in epithelial tissues (Benjamini *p*-value 2.7 × 10^−12^, Supplementary Table S12). It is noteworthy that the up-regulated genes in HGG tumors are significantly enriched on chromosomes 2, 8, 16, 20, and 22 (Benjamini *p*-value < 1.4 × 10^−4^). Furthermore, HGG tumors have higher number of RDG than in LGG tumors (*p* = 2.8 × 10^−13^, Figure 3A). The high number of differentially expressed genes and the functional and chromosomal enrichment of these genes indicate essential distinct genomic and transcriptomic profiles of LGG and HGG tumors.

For CNV data, we compared the number of AG in the LGG and HGG tumor subclasses. The results revealed that the difference in AG between LGG and HGG is significant (*p* = 3.7 × 10^−16^, Figure 3B). Furthermore, these patterns were also observed when deleted or amplified genes were analyzed separately (Supplementary Figure S5A). In all cases, there were more AG in the HGG than in the LGG tumors (median HGG: 3,565 genes per sample; LGG: 1,875 genes per sample).

**Figure 4:**
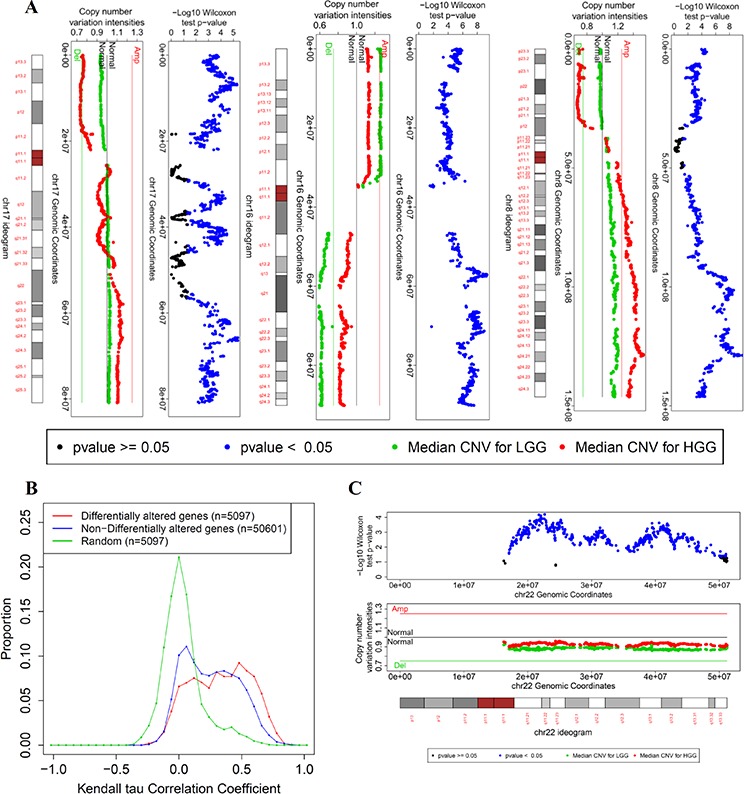
Copy number variation visualization of few chromosomes in which the differentially altered genes between LGG and HGG are enriched **A.** Copy number variation for chromosomes 8, 16, and 17. For each chromosome, three bars are shown: - The upper bar is a plot of the negative log *p*-value of the Wilcoxon test per gene against its transcription start site. The Wilcoxon test assesses the difference in CNV profile between LGG and HGG tumors for each gene. -The middle bar is the median values of the CNV signal intensities of LGG (green) and HGG (red) tumors per gene against its transcription start site. - The lower bar is the ideogram of the corresponding chromosome (centromere in red). **B.** The distributions of Kendall's tau correlation coefficients between CNV and corresponding gene expression of differentially altered genes between LGG and HGG tumors (red), non-differentially altered genes (remaining genes in the genome, blue), and a random match between the CNV profile and gene expression profile as a control distribution (n represents number of different combinations of matching the CNV profiles of genes with their expression profiles of multiple probesets). **C.** copy number variation visualization of chromosome 22.

Next, DAG analysis between LGG and HGG revealed 1,858 DAG (1,432 protein-coding, 347 ncRNA, 61 pseudo, 17 snoRNA, and 1 snRNA, Supplementary Table S13) enriched in a few chromosomes (Table 2D). Specifically, 52% of the DAG (971 of 1,845 genes) are located on chromosome 16. Visualization of the copy number variation status across the chromosome arms showed that in LGG, there is a gain of 16p and deletion of 16q whereas HGG tumors showed gain of 8q and loss of 8p and 17p (Figure 4A). Our results provide plausible evidence to support the hypothesis that LGG and HGG tumors are distinct at the genotype level. In particular, the deletion of 16q in the LGG tumors and the lack of deletion of 16q in the HGG tumors support the model of independent tumor progression into low or high grades. It is noteworthy that both DAG and DEG between LGG and HGG tumors share the enrichment in chromosome 8 and 16 (Table 2D, Supplementary Table S12).

Based on our analysis of somatic mutation profiles, we found that LGG tumors have significantly fewer mutations than HGG tumors. Figure 3C shows a significant difference between LGG and HGG tumors (Wilcoxon *p*-value = 3.8 × 10^−13^). The three types of mutations (missense, nonsense, and silent) show the same trend in the difference in mutations per sample across genetic grades (Supplementary Figure S5B).

The mutation status of *TP53* and *PIK3CA* show a significant correlation with the new classification into two major genetic classes (Fisher's exact test *p*-value for *TP53* = 8.3 × 10^−15^, and for *PIK3CA* = 6.1 × 10^−7^). For *TP53*, the frequency of mutations in this gene consists of the 10% (14/130) in the LGG tumors and the 48% (137/284) in the HGG tumors. This correlation is positive regarding to HGG. Inversely, for *PIK3CA* the frequency of mutations in the gene consists of the 48%(62/130) in the LGG tumors and 23%(67/284) in the HGC tumors, suggesting the negative correlation relatively HGC. Other 10 genes also show significant correlation with the genetic grading into two major classes (Fisher's exact test *p* < 0.05) where 2 genes (*MUC4, TTN*) are highly mutated in HGG tumors whereas 8 genes (*CBFB, CTCF, MAP3K1, CHD8, DYSF, DNAH1, MAP2K4, GATA3*) are highly mutated in LGG tumors (Figure 3E). Specifically, the high mutation rate of the genes in specific regions of LGG tumor cells with respect to HGG tumor cells supports the independence of the oncogenic pathways hypothesis for LGG and HGG tumors.

### DNA copy numbers of the differentially altered genes are strongly associated with their corresponding gene expression profiles

To assess the mechanistic role of DAG in cancer progression, we analyzed the effect of the CNV of each gene on its gene expression profile. A correlation analysis was conducted between the mRNA profile and the corresponding CNV profile for each gene (see Methods). Interestingly, RNA expression and corresponding CNV were significantly correlated for approximately 52% of the DAG (FDR <0.01, 976 of 1,845 genes). Moreover, the DAG (1,845 genes) have stronger correlation with their gene expression profile compared with non-differentially altered genes (non-DAG). Non-DAG, (*n* = 23,327) and randomly matched copy number/expression (background/control) (Figure 4B). Wilcoxon test shows a significant difference between the correlation coefficients of the DAG (median = 0.34) and non-DAG (median = 0.24) (*p* = 4.3 × 10^−105^); the DAG tend to have stronger positive correlations which reflect the importance of the CNV of these genes in driving their gene expressions that lead to functional distinction between LGG and HGG tumors.

### Chromosome 22 copy number variation is a novel indicator of LGG and HGG independence

Generally, a loss of genetic material in low grade tumors but not in high grade represents the striking evidence for the independence of the low- and high-grade oncogenic pathways (e.g. 16q loss). In addition to the loss of 16q in low-grade tumors, 22q shows low CNV signal intensities for LGG tumors compared with HGG tumors. Although the median values of CNV signal intensities for LGG tumors do not pass the threshold of copy number loss, the difference in copy number between LGG and HGG is significant. This difference is notable for genes located downstream of the centromeric region and at the sub-telomeric region (Figure 4C). Generally, LGG tumors have a lower 22q copy number than do HGG tumors (Wilcoxon test *p*-value = 4 × 10^−179^), as shown in a cumulative distribution of all of the 22q genes CNV intensities in Supplementary Figure S7. Collectively, observed patterns of 22q CNV alterations provide plausible evidence to support the hypothesis that the oncogenic pathways related to the LGG and HGG gene expression phenotypes are independent.

### DNA copy number variation reflects sub-classification of HG2 tumors

We studied the discriminative potential of the CNV for classifying histological grades. DAGs between HG1 and HG3 tumors were determined using the same criteria used previously for the selection of DAG. We obtained 1,486 genes localized on 16p, 16q, 17p, 8p, and 8q. Next, we selected the top gene from each chromosome arm that has the minimum Wilcoxon test *p*-value as a representative marker for its chromosome arm CNV event. Therefore, 5 genes (*LOC286114* for 8p, *MYC* for 8q, *POLR3E* for 16p, *HERPUD1* for 16q, and *ZNF18* for 17p) were selected for subsequent class discovery analysis. Using SWS algorithm, the classifier was trained using HG1 and HG3 tumors. Similar to the gene expression data, HG3 tumors were shuffled and divided into 7 non-overlapping groups, and 7 training-prediction subsets were performed. The average classification accuracy was 77 ± 4.3%. HG2 tumors were sub-classified into HG1-like and HG3-like tumors in each training-prediction subset. Each HG2 sample was assigned to a new subclass according to the consensus classification in all 7 classifiers. According to these criteria, 93 samples were classified as HG1-like (67 of 93 samples matched with gene expression-based HG1-like samples), and 73 samples were classified as HG3-like (42 of 73 samples match with gene expression-based classified HG3-like samples). Sixteen samples showed intermediate assigning probabilities and were considered HG2. We have found significant positive agreement between the classifications of HG2 tumors based on gene expression and copy number variation data (Cohen's kappa coefficient = 0.32, *p*-value = 7.4 × 10^−5^, Supplementary Table S14). These results indicate that our classification of IDC tumors into LGG and HGG tumors can be achieved at genomic and transcriptomic level. However, the agreement between the mRNA-based and DNA-based classification is moderate, perhaps due to the differences in mechanisms of regulation at these two levels of molecular organization of gene expression.

### The LGG and HGG grading classification is associated with the differential expression of stem cell genes

To relate the grading classification with tumor stemness, we investigated whether the genes associated with stem cells were enriched among DEG between HG1-like and HG3-like samples. We used Cancer Genome Anatomy Project data, for which serial analysis of gene expression (SAGE) was used to study genes expressed in 21 embryonic stem cell lines. Interestingly, all gene lists related to the 21 stem cell were over-represented in the up-regulated genes in HG3-like tumors (Benjamini *p*-value <8.3 × 10^−24^, Supplementary Table S15A). Moreover, we checked the discriminative capability of the stemness-associated genes in the sub-classification of HG2 samples. We extracted the common genes expressed in all 21 stem cell lines independent from the grading associated genes. We obtained 106 genes that are expressed in all the studied 21 stem cell lines (Supplementary Table S15B). Subsequently, we used unsupervised hierarchical clustering on the TCGA gene expression profile of these genes using Euclidean distance for similarity measurement and average linkage as agglomerative method. The results showed a formation of two major clusters. We found strong correlation between these two clusters and the grading classifications of LGG and HGG (Cohen's Kappa correlation = 0.57, *p* = 3.3 × 10^−31^, Supplementary Table S16, Supplementary Figure S8). The concept of distinct precursors of LGG and HGG tumors provides a plausible explanation for these results.

## DISCUSSION

Our integrative analysis and intrinsic subtype distributions within HG2 tumors demonstrate the strong molecular distinction between HG1-like and HG3-like tumors and their comparable genetic profiles with HG1 and HG3 tumors, respectively. Based on these similarities, we considered HG1-like and HG1 tumors to be LGG tumors, and similarly, we considered HG3-like and HG3 tumors to be HGG tumors. We tested the hypothesis that LGG and HGG tumors are the two major genetically predetermined classes of breast IDC and that they have independent oncogenic pathways. Similarly, the distinction between LGG and HGG tumors was supported based on integrative data analysis. We found 4,879 protein-coding genes that were differentially expressed between LGG and HGG tumors, which represent 23.2% of the total protein-coding genes annotated in the genome. This systemic shift in the transcriptomic program implies that there are independent oncogenic pathways that dichotomize IDC tumors into these two subtypes. These two oncogenic pathways are distinguished primarily in cell proliferation and cell adherence phenotypes.

Because mRNA expression is temporally regulated during the cell cycle and differentiation, justifying molecular grading at the DNA level is an essential step to understanding tumor heterogeneity and the independence of LGG and HGG tumor progression. While an association between DNA copy number variation (CNV) and histological grades is expected because of the inclusion of the mitotic index and nuclear polymorphisms in histological grading systems [54], this association has not been explored using large cohorts and high-resolution techniques. DNA copy number variations and point mutations are the major genetic changes that drive tumor development. Generally, we show that the number of altered genes is much higher in HGG tumors than in LGG tumors. The DAGs discriminating between LGG and HGG tumors are enriched in specific chromosomes where chr16 is the major contributor. Five major events were observed, 16q loss, and 16p gain in LGG tumors and 8p, 17p loss and 8q gain in HGG tumors. Our gene-centric based copy number variation analysis helps to highlight candidate genes of which copy number alterations give a survival advantage to tumor cells during tumor evolution.

The frequent loss of 16q in LGG tumors is another line of evidence that supports the improbable progression between LGG and HGG tumors. Regaining lost genetic material is unlikely, and thus the inter-grades progression is improbable. However, it was reported that the loss of 16q in HG3 tumors is followed by mitotic recombination [18]. This recombination makes 16q loss ostensibly less frequent in high grades, especially when allelic imbalance is not taken into account during copy number variation analysis. However, a high allelic imbalance of 16q in low grade tumors was observed previously based on three microsatellite markers [55]. Moreover, we observed that 22q-related genes show an overall low copy number status in LGG tumors with respect to HGG tumors. This observation is similar to that observed in 16q and may act as a supporting evidence of the independent oncogenic pathways too. The strong correlation of the DAG with their gene expression, in contrast to the non-DAG, reflects the importance of CNVs in driving the distinction between LGG and HGG tumors. Therefore, the DAGs are a shortlist of candidate genes and genome loci associated with the independent oncogenic pathways in LGG and HGG tumors. Collectively, observed CNV alterations provide a genomic basis for future development of diagnostics and prognostic assays.

For point mutations, general comparison of the number of point mutations in LGG and HGG tumors shows a significantly different mutation profile. Overall, LGG tumors have fewer mutations than HGG tumors. Specifically, the most mutated genes, *TP53* and *PIK3CA*, have mutations counts positively and negatively correlated with genetic grades respectively. Our analysis demonstrated that a relatively higher count of *PIK3CA* mutations is associated with HG1-like tumors. As *PIK3CA* mutations frequently occurs in IDC and are known to activate the PI3K/AKT/mTOR pathway, these mutations could be considered as potential predictive biomarkers of HG1-like tumors. High mutation rate of *PIK3CA* in LGG with respect to HGG indicates that *PIK3CA* hotspot mutations could have the potential to predict intrinsic tamoxifen resistance in the adjuvant treatment of LGG ER^+^ BC patients. The testing of this hypothesis should be the interest of future studies. In addition, LGG and HGG showed differences in mutation counts of *MAP3K1* and *MAPK2K4* that are functionally linked with *PIK3CA*. Interestingly, 9 of 12 top frequently mutated genes, (*PIK3CA, GATA3, MAP3K1, MAPK2K4, CBFB, DNAH1, CTCF, CHD8,* and *DYSF*; Figure 3E), also demonstrated significantly higher mutation counts in LGG with respect to HGG IDC cells. These findings support the hypothesis of independence of the oncogenic pathways of LGG and HGG tumors.

We observed moderate but significant differences in CNV levels between HG3-like and HG3 tumors. These observations may be artificial because the multiple grading systems used to evaluate the histological grades of the TCGA cohort could introduce some bias into the quantitative determination of HG3 tumor classification in addition to the subjectivity of all these grading systems. Interestingly, these DNA variations do not result in any functional transcriptomic discrimination between HG3 and HG3-like sub-classes of IDC. However, observed differences between HG3 and HG3-like IDC tumors could reflect actual patho biological differences which should be a topic of future studies.

The ongoing open question where the functional heterogeneity of IDC is due to the cell of origin or accumulation of mutational events is still unanswered [56, 57]. The measure of cell differentiation in grading systems makes the association of stem cells with histo-pathological grades self-evident. However, this association has been studied in only a limited number of studies [58, 59]. An enrichment of cancer stem cells (CSC) in high histological grades has been shown with respect to low grade [59]. Several stem-cell-based models of cancer initiation and progression have been suggested for different intrinsic subtypes of IDC. However, data are controversial and further studies are needed for the specification and validation of these models [56, 57]. It was argued that good-prognosis ER^+^ tumors could initiate via clonal selection and have limited number or no CD44^+^/CD24^−^ cells. However, poor-prognosis ER^+^ tumors could initiate from ER^+^ stem or progenitors cells and expand to have a mixture of ER^−^/CD44^+^/CD24^−^ and ER^+^/CD44^−^/CD24^+^ cells [56]. Collectively, based on our observation of strong expression differences between LGG and HGG tumors for genes associated with embryonic stem cells, the different frequency of ER loss between LGG and HGG tumors (Table 1), and the distribution of intrinsic subtypes within them, it can be assumed that LGG tumors originate and progress depending on the clonal evolution of normal epithelial cells, whereas HGG tumors originate from stem/progenitor cells and progress via clonal evolution to multiple subtypes to include ER^+^ and ER^−^ tumors.

**Figure 5:**
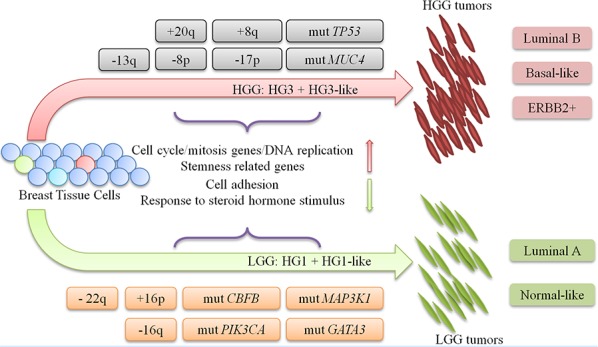
progression model for LGG and HGG tumors IDC tumors progression model shows the major genetic events that dichotomize and characterize each oncogenic pathway of LGG and HGG tumors. DEG: differentially expressed gene. CSC: Cancer Stem Cell. +: DNA copy number gain. −: DNA copy number loss. mut: DNA point mutation.

Thus, we provided for the first time an integrative characterization of LGG and HGG classes of IDC tumors by gene expression, CNV and mutation data analyses. We presented several lines of evidences that support concept of independent origin and independent oncogenic pathways in LGG and HGG classes, as well as the improbability of inter-grade progression. The distinct molecular events leading to either LGG or HGG tumors are outlined in the tumor progression model shown in Figure 5.

Our 22g-TAG signature with known cell cycle function, clinical measures of cancer proliferative capacity such as Ki67 staining and pathological mitotic index, could be used in the parallel or single assays.

High tumor grade is associated with decreased overall survival [60], but it also known that it predicts increased response to neoadjuvant chemotherapy [61, 62]. Consequently, we can hypothesize that IDC with LGG and HGG would also show decreased and increased response rates to the chemotherapy respectively.

According to our classification, in HGG IDC many hundreds genes of cell cycle, mitosis and DNA replication are overexpressed which are typically associated with higher sensitivity to neoadjuvant anthracycline- and taxane-based chemotherapy in both ER-positive and ER-negative IDC subsets [63]. Consequently, we expect that HG3-like IDC in HG2 (and HGG tumors) would have high response to the conventional chemotherapy targeting the pathways related to rapidly proliferative epithelial cells.

In contrast LGG tumors are expected to be less suitable for treatment as high-aggressiveness tumors. Therefore, they could be more suitably treated with agents that target other growth-related requirements of tumors, such as the mTOR pathway that mediates mRNA translation and increase genome instability of tumor cells and initiate their apoptosis. Further examples include agents that mediate the growth of blood vessels that provide blood supply to tumors (such as bevacizumab) or hormone-related growth signaling pathways (estrogen signaling pathways in ER+ tumors) such as tamoxifen.

Among 22g-TAG genes, it is important to highlight NAT1 and MELK as ‘druggable’ targets which are highly expressed in LGG and HGG tumors, respectively, with respect to normal tissue. It was shown that NAT1 can be inhibited efficiently by Rhod-o-hp with minimal cell toxicity or by iRNA to decrease cell growth and invasiveness [64, 65]. In addition, MELK was successfully targeted by OTSSP167 compound and demonstrated a suppression of mammosphere formation in breast cancer cells and growth suppression of xenograft studied in multiple cancer types in mice [66–68]. Therefore, it should be important to consider *NAT1* and *MELK* genes and their products as the targets in the therapeutic plans for LGG and HGG tumor separately.

Moreover, four genes of 22g-TAG (*BUB1, KIF2C, UBE2C,* and *CENPN*) in addition to *CDC20* (from 22g-TAG network) are among the 10 genes that determine the responsiveness of tumors to chemotherapy recently identified [69].

Neoadjuvant chemotherapy (NAC) can cause tumor shrinkage, which enables a proportion of patients with large tumors to be eligible for breast conservation surgery (BCS). This increases the BCS rate in comparison to adjuvant chemotherapy only [70]. In such cases, our genetic grading classification could potentially be useful for prediction of patients' eligibility to NAC.

## CONCLUSION

Our methodological approach of the integrative data analyses of histologic grads rejects the old hypothesis of the inter-grade progression from HG1 toward HG3 tumors of IDC. Alternatively, the IDC patient population dichotomization based on the multiple key cancer-associated molecular factors and mechanisms, were characterized by the 5691 DEGs and by the 1858 DAGs reported in this study. Collectively, this study strongly supports our hypothesis of the genetically-defined low- and high-grade tumors corresponding to two oncogenic pathways independently governing the progression of LGG and HGG IDCs. Our grading delineation could help to narrow the IDC biomarker space, specify essential characteristics of the two main IDC classes. Eventually, our concept and findings have the potential to impact on patient care, diagnostic and treatment decisions to develop rational strategies for future personalized molecular targeting of IDC.

## MATERIALS AND METHODS

### Data source and preprocessing

Clinical information and gene expression data for the Uppsala, Stockholm, Singapore and Marseille BC cohorts were obtained from the NCBI/GEO database series GSE4922, GSE1456, GSE4922 and GSE21653, respectively.

The Cancer Genome Atlas (TCGA) data is available at multiple levels of preprocessing steps for each data type. We used gene expression, DNA mutation, and DNA copy number variation (CNV) data for IDC. Each data type was downloaded at preprocessing level appropriate for our subsequent analysis [51].

Level 2 TCGA Gene expression data, profiled using Agilent Technologies G4502A, was downloaded. Data was already normalized against Stratagene Universal References RNA, and then Lowess normalization was applied for each probeset (*n* = 90,797). We restricted our analysis to Invasive Ductal Carcinoma (IDC) of no special type (NST), which constitutes 82% of the cohort (481 of 590 samples). Among the 481 samples, there are 48 normal samples from tumor adjacent tissues, and 3 unknown histological grades. The distribution of the histological grades of the remaining 430 samples is uneven (HG1 = 32 (7.4%), HG2 = 183 (42.6%), HG3 = 215 (50%) samples). The information about histologic grades has been manually extracted from the available unanimous histologic reports of TCGA database.

Level 1 CNV data corresponding to our 430 IDC samples was downloaded from TCGA (upon General Research Use access approval). This subset of samples consists of 860 samples (430 tumor/normal pairs). CEL files were imported into Partek^®^ Genomics Suite^™^ software for the extraction of aberrant genomic regions in any tumor sample with respect to its corresponding matched normal DNA sample extracted from blood (paired analysis). A circular binary segmentation algorithm was chosen to infer the regions with genomic aberrations using the default parameters (10 minimum markers in the detected region and *t*-test *p*-value < 0.001 between the altered region and its neighbor region). Genes included in each reported genomic region were extracted using Refseq data. The data was then converted into a two-dimensional matrix in which the rows represent the genes, the columns represent the samples, and the data values represent the mean value of CNV marker intensities of the reported aberrant region that harbors a given gene in a given sample.

Level 2 DNA somatic mutation data were downloaded from TCGA identified using exome sequencing. The mutation annotation file (MAF) contains information about the mutated genes, mutation genomic coordinates, type of mutation, and genotype calls of the tumor and reference normal samples for each patient. Only 418 samples are common with the chosen 430 IDC samples. Data were converted into a two-dimensional matrix in which the rows and columns represent the genes and samples, respectively, and the data points represent the number of distinct mutated sites of a given gene in a given sample.

### Prediction analysis of microarray (PAM)

PAM is a modified nearest-centroid method used for features selection and class prediction analyses [34]. In this work, we used it for dimensionality reduction to obtain most informative and representative features from the entire set of microarray probesets that discriminate between HG1 and HG3 tumors. PAM was implemented via the “pamr” R package.

### Statistically weighted syndrome (SWS)

SWS is a statistics-based voting class prediction and feature selection method. It selects the most informative variables (prediction features), categorizes them and tests the stability of the classification border of a feature domain of the training set based on sampling and a leave-one-out procedures [26, 35]. We used the features resulted from PAM analysis to sub-classify HG2 samples into HG1-like and HG3-like tumors based on SWS algorithm. SWS was implemented in Recognition software (http://www.solutions-center.ru/index.php?sct=prod)

### Normalization of probeset expression and identification of reference-deviated genes per sample (RDG)

For each TCGA IDC tumor sample, we normalize the expression of each probeset with respect to the reference normal expression for that same probeset. This reference is represented by its median expression in the 48 normal samples. The normalized probeset expression relative to the reference normal dataset can be referred to as the fold-change. The data was already normalized by Lowess normalization for all chips. Variation of coefficients for 25%, 50% and 75% quartiles for all chips are 0.059, 0.014, and 0.054 respectively.

For each TCGA IDC tumor sample, to identify RDG, fold change criteria ≥ 1.25 or ≤ 0.75 were used. The number of RDG for each TCGA IDC tumor sample can be calculated independently and compared across the genetic grade subgroups.

### Copy number variation visualization

Median values of the CNV intensities for LGG and HGG tumors and Wilcoxon test *p*-value for each gene were plotted against the genomic coordinates of its transcription start site. A chromosomal region is considered altered if the median values of its genes pass one of the global thresholds of loss or gain (i.e., greater than 50% of the patients undergo the CNV event).

### Identification of differentially expressed genes

A two-tailed Wilcoxon test was used to assess the significance of the differential expression, and Benjamini-Hochberg (FDR) correction was used for multiple hypothesis testing. Differentially expressed probesets were selected based on fold-changes (FC ≥ 1.5 or FC ≤ 0.75) and statistical significance (FDR < 0.01).

### Association analysis of gene expression and copy number variation

Kendall tau correlation was utilized to study the association of CNV and corresponding mRNA expression for each gene. CNV and mRNA expression data matching was performed using Agilent 244K Custom Gene Expression G4502A-07–3 annotation data provided by TCGA data portal.

### Functional enrichment and gene ontology analysis

The Database for Annotation, Visualization and Integrated Discovery (DAVID) [71] tool was used to identify the top enriched biological processes among the differentially expressed genes through the Gene Ontology (GO) annotation database. Input of unique Entrez genes IDs was compared with a background gene list constitute all the genes in the genome using Hypergeometric test. Functional annotation chart constitutes of molecular functions, biological processes, cellular components, KEGG pathways, tissue expression, and chromosome number was reported.

### Network analysis of 22g-TAG genes

The MetaCore tool (Thomson Reuters, St. Joseph, MI, USA) was used to build the genes network associated with 22g-TAG genes (https://portal.genego.com/).

### Hierarchical clustering (HC) and heatmap visualization

Multi-experiment viewer version 4.9.0 was utilized to conduct HC and heat map visualization of numerical matrices. Euclidian distance and average linkage agglomerative method were used to achieve HC.

### Data-driven prognosis analysis based on gene expression profile

Survival analyses were conducted using a data-driven grouping (DDG) algorithm which relies on Cox-proportional hazard regression model to fit the patients' survival times to gene expression data (see supplementary material in [73]). It searches for the best cutoff of the expression of a given gene that maximizes the separation of the survival curves of the patients into high- and low-risk groups for each gene. DDG has been successfully used in prognosis of breast, glioblastoma and ovarian cancer patients [72, 73].

Univariate and Multivariate analyses were conducted using the “survival” R package version 2.37–7.

### qPCR based validation of 22g-TAG genes

Total RNA samples of 84 IDC patients were obtained from OriGene (patients' clinical parameters are summarized in Supplementary Table S17A). The concentration of the RNA was provided by OriGene, reconfirmed using a Nanodrop^®^ spectrophotometer, and normalized. cDNA synthesis from 250 ng total RNA was conducted using a QuantiTect^®^ Reverse Transcription Kit based on random hexamer and Oligo (dT) primers. qPCR experiments were conducted in 96-well plates using the QuantStudio™ 6 Flex Real-Time PCR System. The KAPA SYBR^®^ FAST qPCR Kit was used for qPCR experiments, and low Rox was used as a passive reference dye. Primers were designed using primer3 (v. 0.4.0) [74], and the specificities of obtained primer pairs were tested computationally using BLAT [75] and in-silico PCR on the UCSC genome browser [76]. The primer pair sequences for 22g-TAG are listed in Supplementary Table S17B. We used β-actin as an endogenous control. The obtained Ct values of all genes were analyzed using the 2^−ΔΔCt^ method [50].

## SUPPLEMENTARY MATERIALS FIGURES AND TABLES





























## Abbreviations

BC: breast cancer
IDC: invasive ductal carcinoma
DEG: differentially expressed genes
DAG: differentially altered genes
RDG: reference deviated genes
LGG: low genetic grade
HGG: high genetic grade
HG (1,2,3): histological grade (1,2,3)
TCGA: The Cancer Genome Atlas
CNV: copy number variation
SNP: single nucleotide polymorphism
SWS: statistically weighted syndrome
PAM: prediction analysis of microarray
