# Supplementary material for: Genome and transcriptome delineation of two major oncogenic pathways governing invasive ductal breast cancer development
Source: Oncotarget. 2015 Oct 10;6(34):36652–74. doi: 10.18632/oncotarget.5543 (PMC4742202; doi:10.18632/oncotarget.5543)
Supplement: Supplementary file 1 [file oncotarget-06-36652-s001.pdf]

SUPPLEMENTARY FIGURES AND TABLES

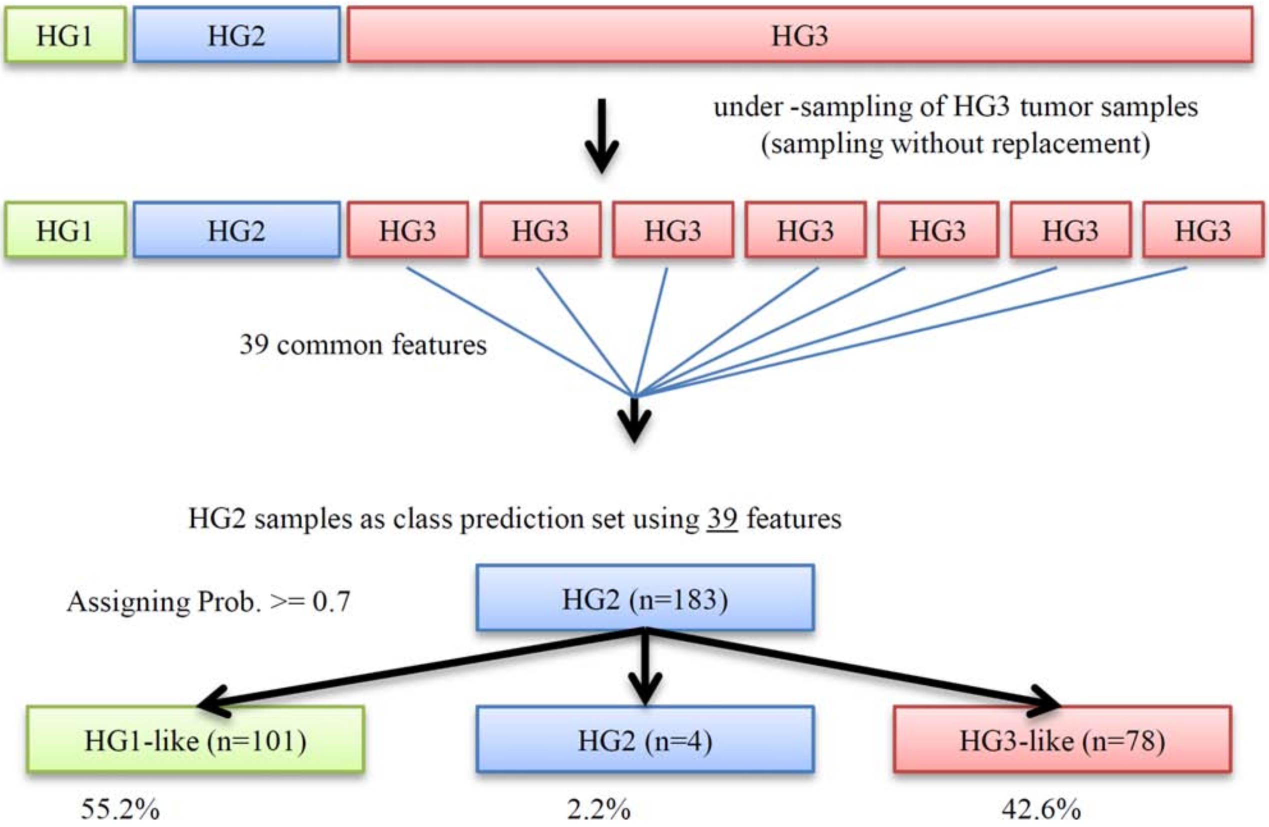

Supplementary Figure S1: under-sampling representation of HG3 tumor samples during pattern recognition analysis. Schematic view of our methodology to overcome the class imbalance in training set. HG3 tumors were shuffled and split into 7 non-overlapping subsets. Each unique subset of HG3 tumors was compared with HG1 tumors to obtain balanced training set during pattern recognition analysis.

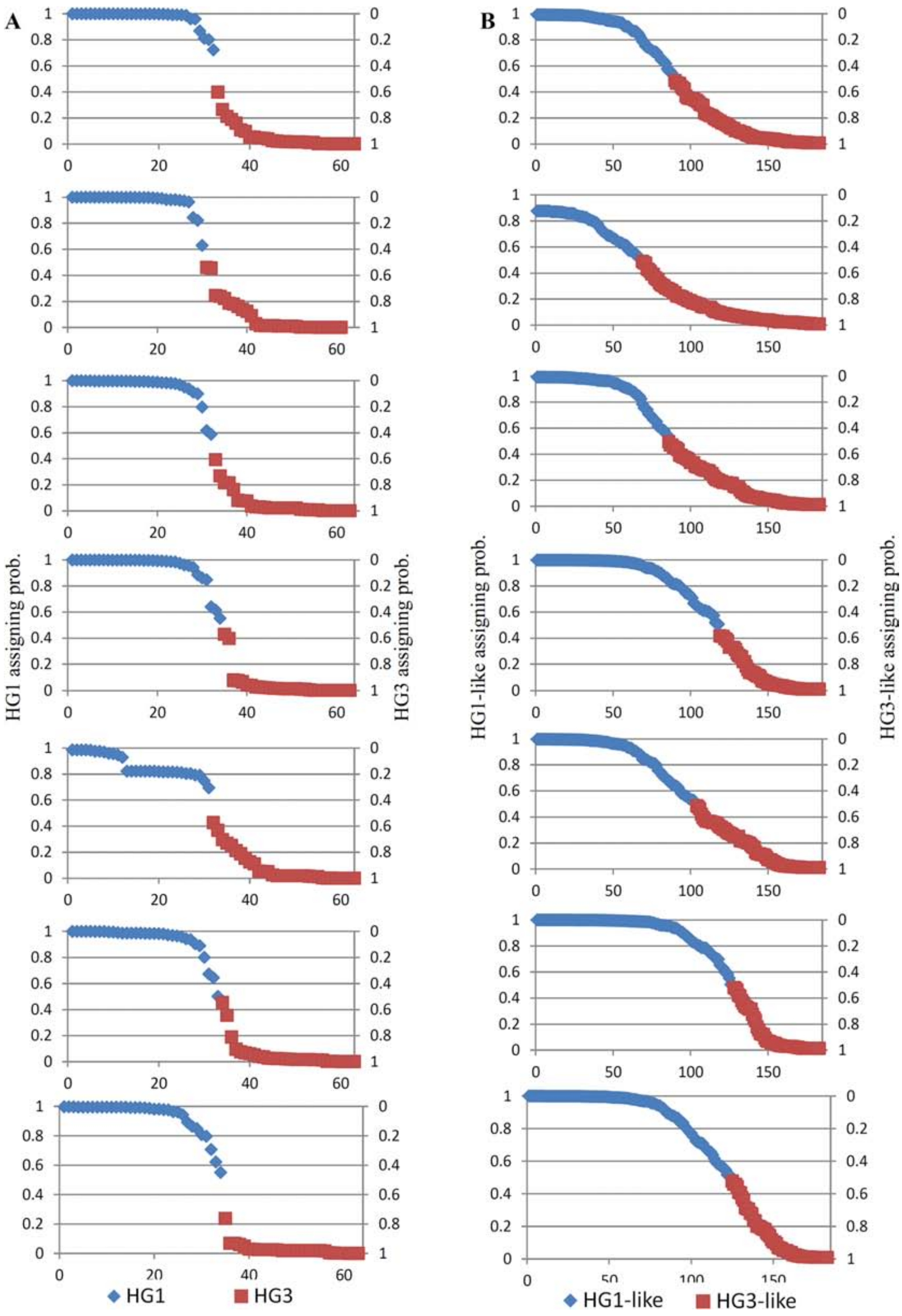

Supplementary Figure S2: SWS derived assigning probabilities of HG1, HG3 and subclasses of HG2 tumors to corresponding genetic subclasses. **A.** the assigning probabilities of HG1 and HG3 tumors to low and high genetic grades during 7 training iterations using 22g-TAG signature based on SWS algorithm. **B.** the assigning probabilities of HG2 tumors to low and high genetic grades during 7 prediction iterations using 22g-TAG signature based on SWS algorithm.

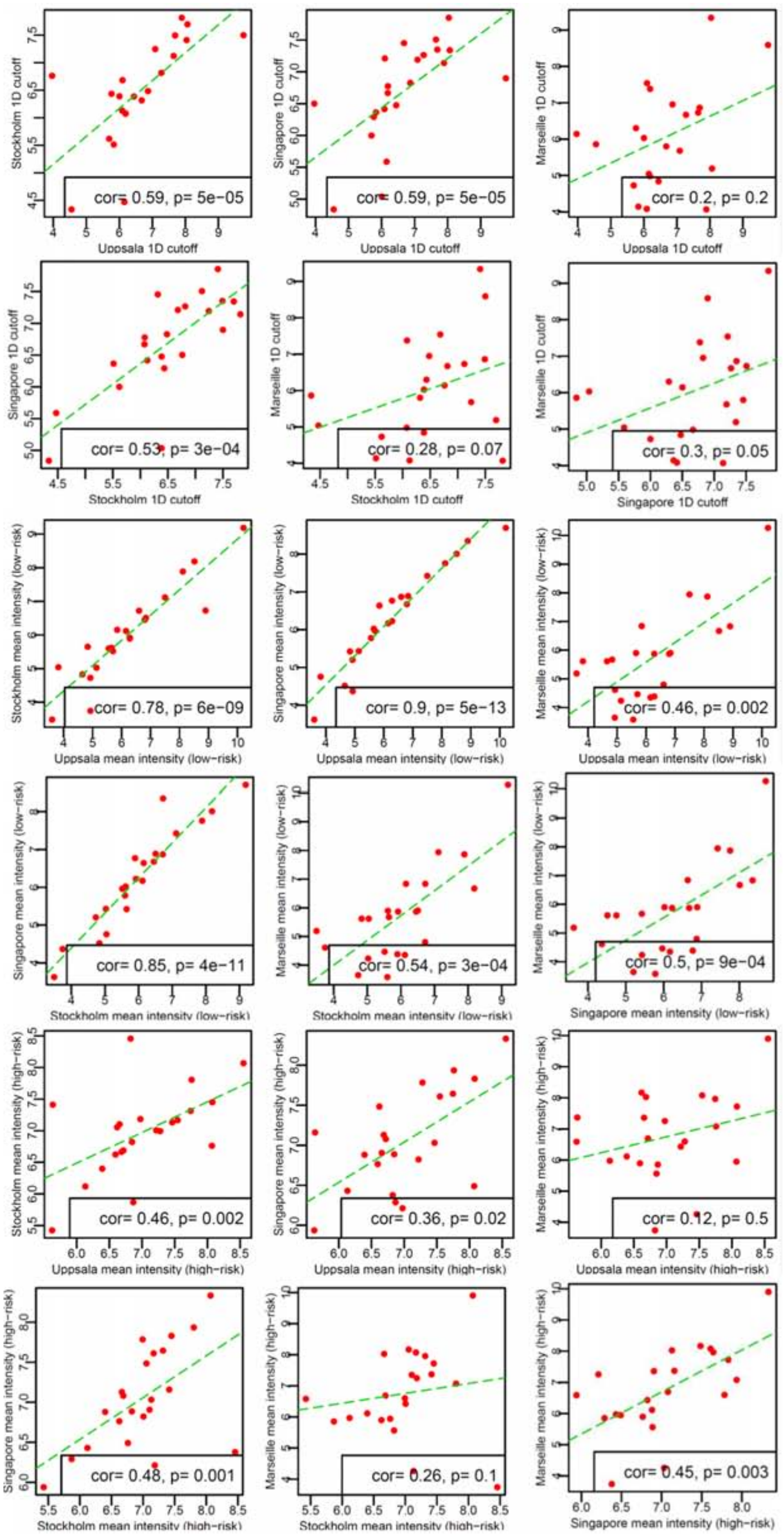

Supplementary Figure S3: scatter plot of the data driven cutoff and mean values of gene expression in low and high risk groups of prognosis prediction analysis of 22g-TAG genes. Scatter plots show the correlations of data driven cutoffs (1D cutoff) of 22g-TAG genes between different cohorts as a test of their reproducibility and robustness. Similarly, for mean values of gene expression in low and high risk groups' tumors. Kendall tau correlation was used to for calculating correlation coefficients and *p*-values.

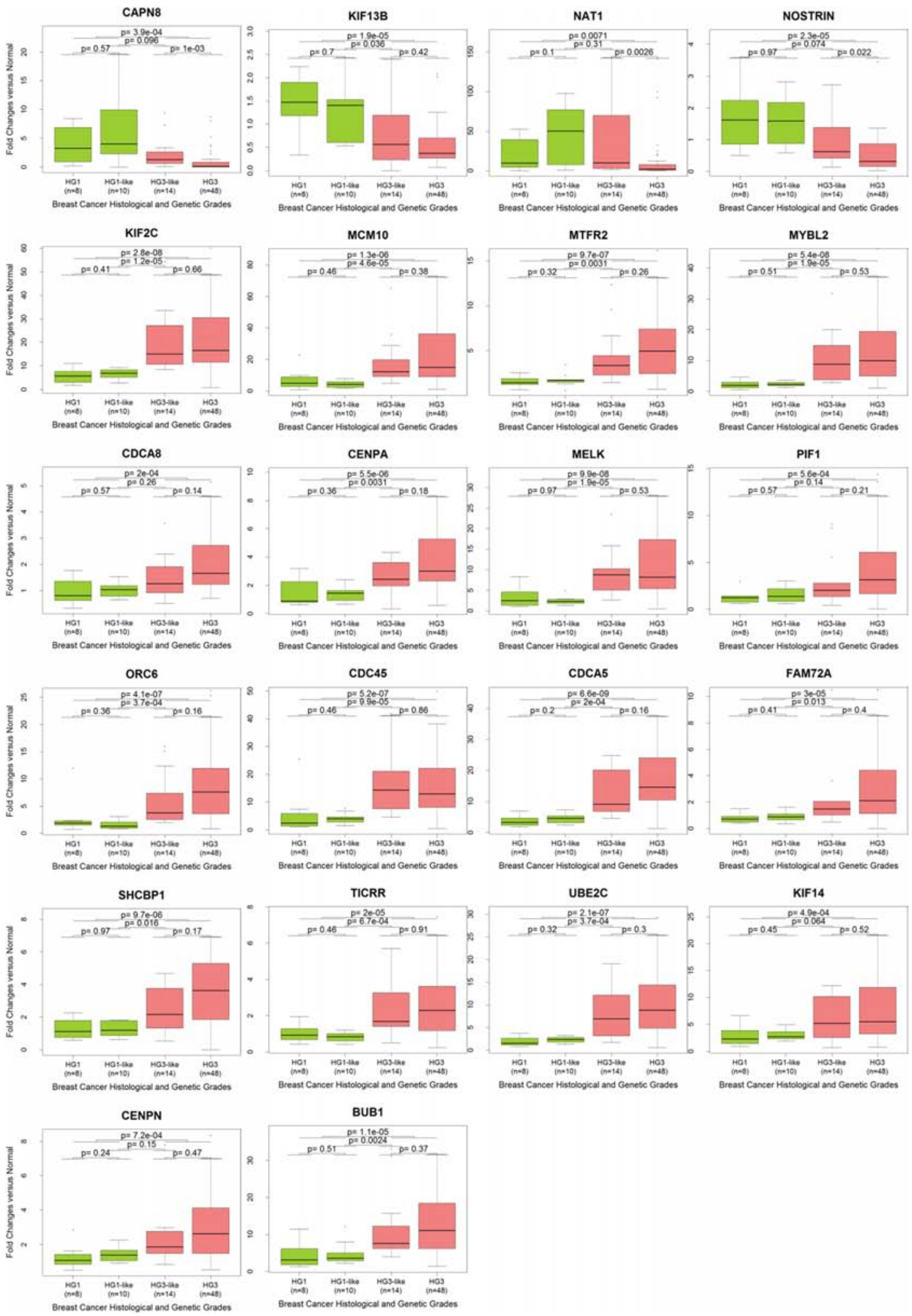

Supplementary Figure S4: Box plot of the relative expression based on qPCR data of 22-TAG genes. Box plots of the relative expression based on qPCR validation data of 22g-TAG genes expression in HG1 (n = 8), HG1-like (n = 10), HG3-like (n = 14) and HG3 (n = 48) tumors of Origene cohort. Two-tailed Wilcoxon test was used to assess the differences in the expression profile between different combinations of histological and genetic grades grades.

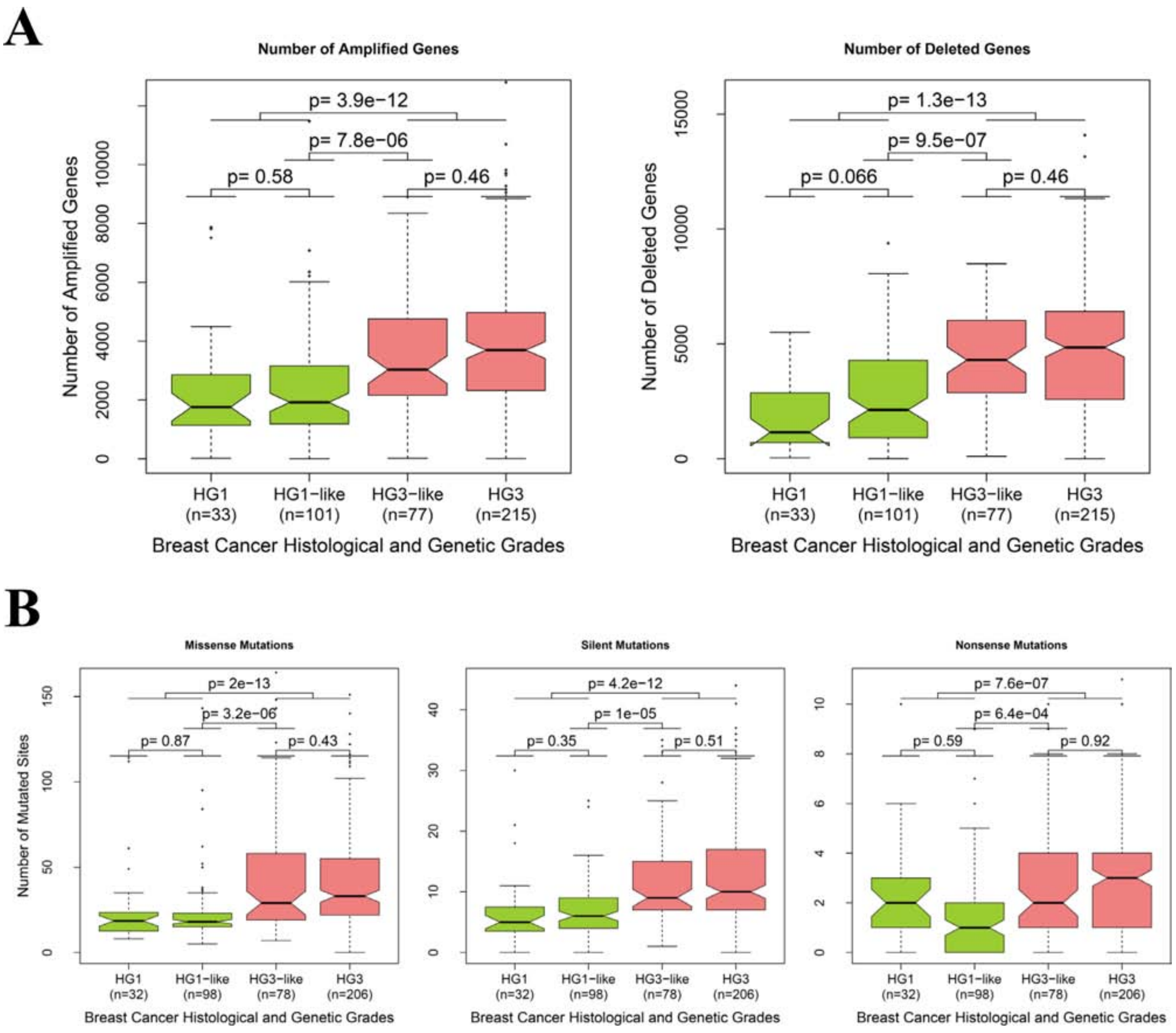

Supplementary Figure S5: box plots of AG and mutation counts per samples for different histological and genetic grades. A. box plots for of the number of amplified or deleted genes per sample separately for different histological and genetic grades. **B.** box plots of the count of missense, nonsense and silent mutations per sample separately for different histological and genetic grades. The difference in the number of altered genes or mutations counts between different combinations of genetic grades was assessed statistically using two-tailed Wilcoxon test.

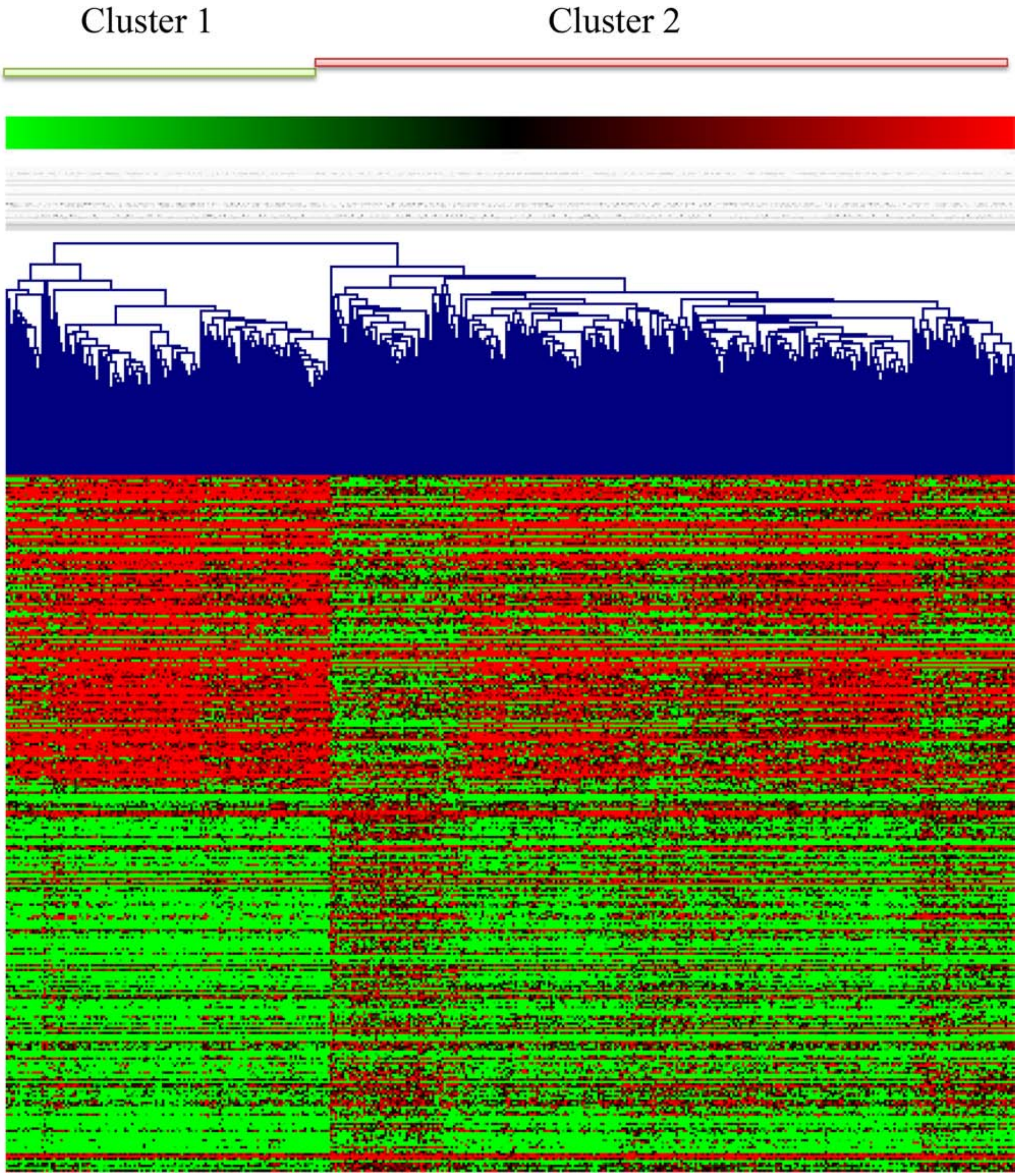

Supplementary Figure S6: Unsupervised hierarchical results. Heatmap of gene expression of differentially expressed genes between HG1-like and HG3-like tumors clustered by unsupervised hierarchical clustering. Euclidean based distance measurement and average linkage agglomerative methods were used for hierarchical clustering.

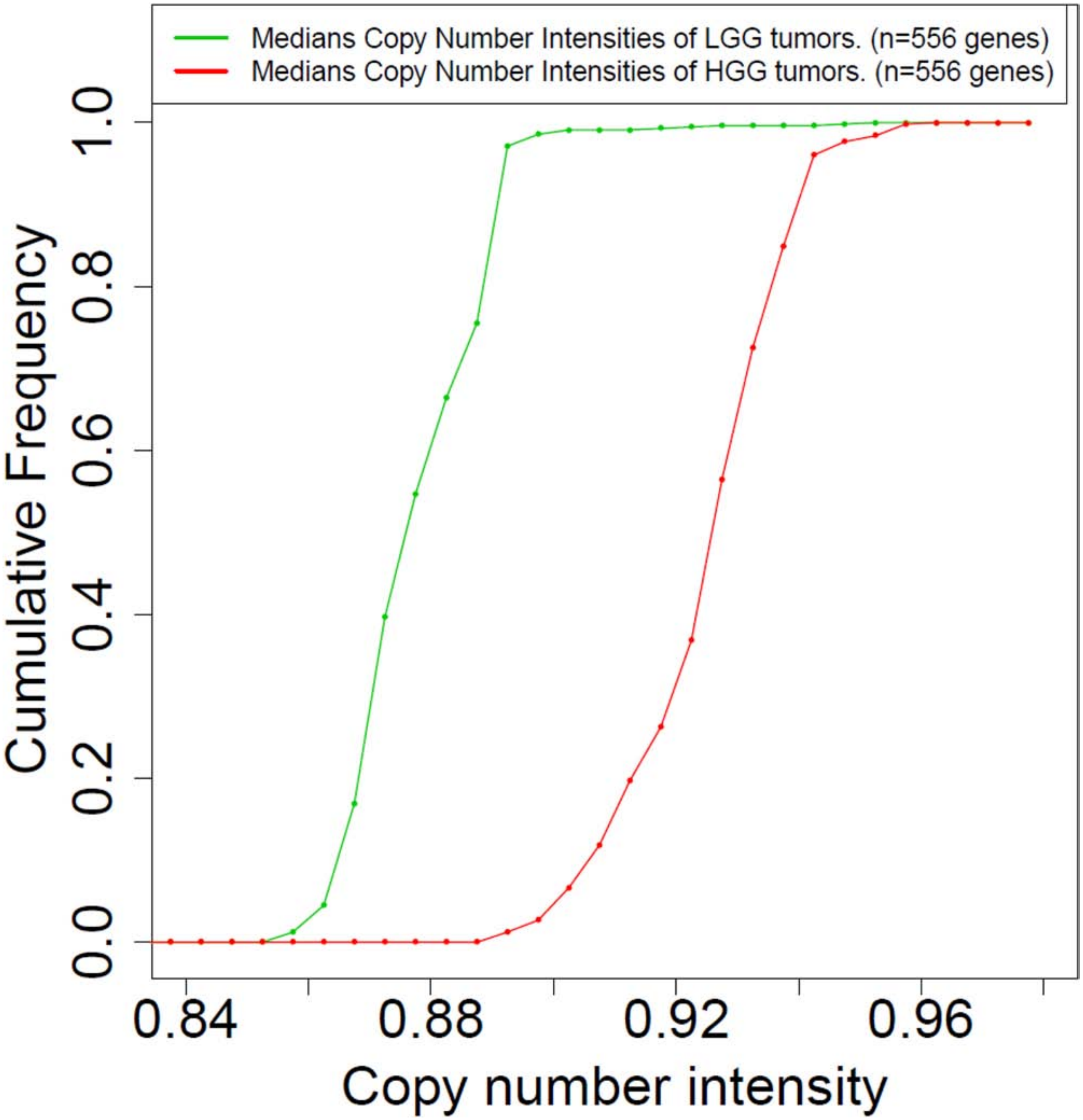

Supplementary Figure S7: cumulative distribution of copy number variation of 22q genes. Cumulative distributions of median values of copy number signal intensities of 556 genes of 22q in LGG and HGG tumors.

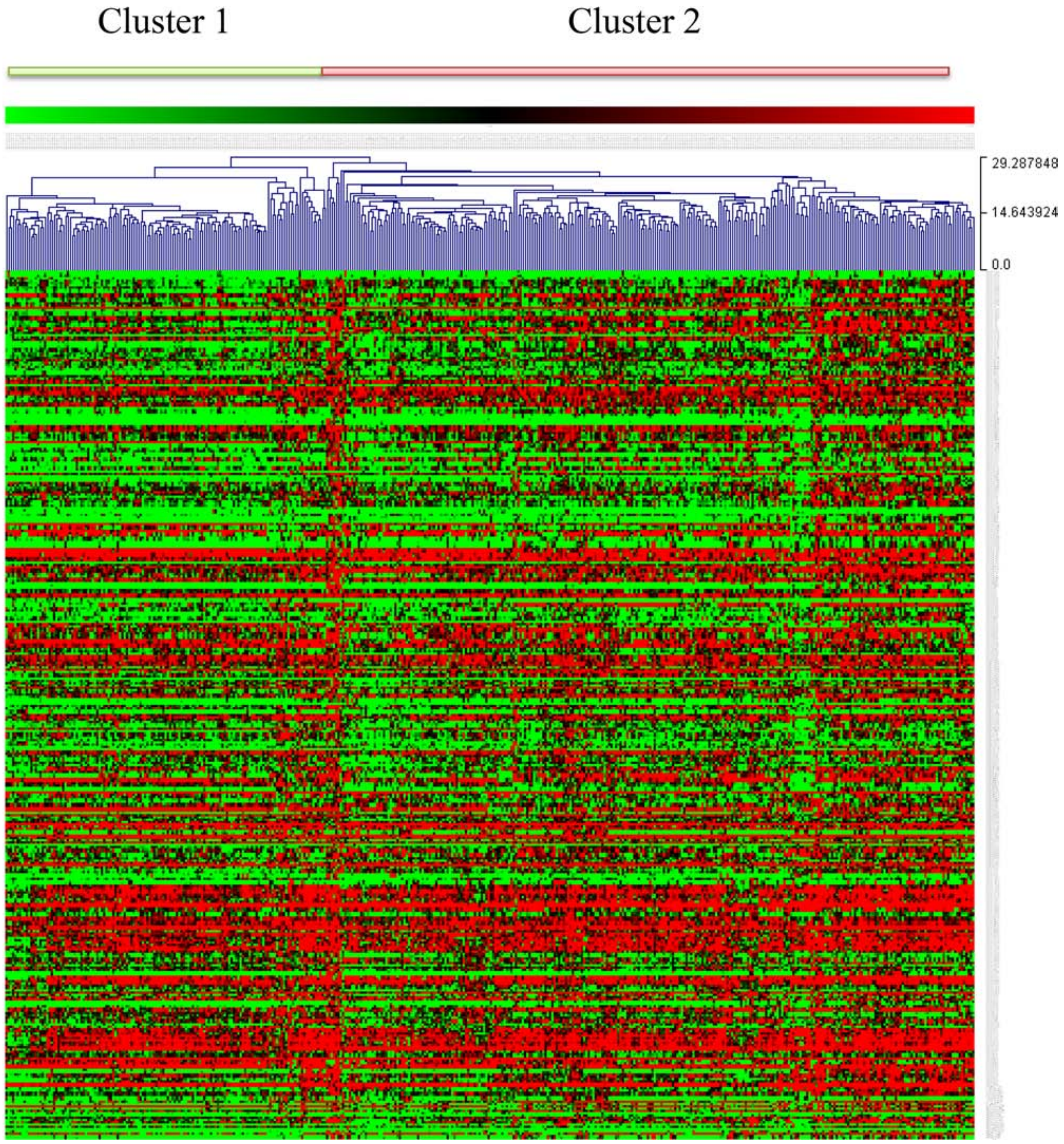

Supplementary Figure S8: hierarchical clustering results performed on 106 genes associated with 21 embryonic stem cells. Heatmap of gene expression profiles resulted from hierarchical clustering of 106 genes expressed in 21 different embryonic stem cells according to SAGE database. Euclidean distance as distance measurement and average linkage agglomerative methods were used for hierarchical clustering.

Supplementary Table S1: Results of PAM pattern recognition analysis applied on HG1 and HG3 tumors. Confusion matrices show training accuracies of seven training sets using PAM classifiers. The seven training sets are results of under-sampling procedure applied on HG3 tumors to overcome imbalance training dataset. PAM classification parameters (Class Error rate; Shrinking threshold; Overall error rate) and the number of top discriminative probesets resulted from each training are shown.

Supplementary Table S2: tumor aggressiveness grading signature. A. The annotation of the 39 probesets corresponding to the 22g-TAG signature for TCGA gene expression level 2, Agilent G4502A_07_3 platform. **B.** The periodicity of the 22g-TAG genes based on experimental data from the Cyclebase database and other published databases. The *p*-values quantify the periodicity and regulation through cell cycle of given gene. The rank represents the rank of gene's periodicity among all periodic genes, where the smaller rank the higher significant periodicity.

Supplementary Table S3: the frequency of 22g-TAG signature genes' occurrence in 72 breast cancer signatures

22g-TAG genes	Number of Occurrences
CAPN8	0
ORC6	1
PIF1	1
FAM72B	3
TICRR	3
SHCBP1	4
CDC45	5
FAM72A	6
NOSTRIN	6
KIF13B	7
KIF14	7
MTFR2	7
MCM10	8
CENPN	9
KIF2C	9
CDCA5	10
NAT1	11
CENPA	12
BUB1	13
UBE2C	13
CDCA8	16
MYBL2	18
MELK	20

The occurrence of 22g-TAG genes in 72 breast cancer related signature including two molecular grading signatures. The number of occurrences: represents the number of reference signatures that contain a given gene of 22g-TAG signature.

Supplementary Table S4: Survival prediction of patients grouping into low- and high- risk subclasses derived using 22g-TAG genes. Survival prediction analysis of 22g-TAG was performed using 1D-DDg method based on gene expression cut-off value estimated using Cox proportional hazards model in four independent cohorts: A. Uppsala, B. Stockholm, C. Singapore and D. Marseille. 1D DDg-defined cut-off: gene expression cut-off corresponding to the most significant separation of the patients into low- and high- risk groups. 1D DDg p-value: log-rank statistic p-value corresponding the optimized 1D DDg-defined cut-off value. C.I.: Confidence Interval. Model design: 1: tumor suppressor-like gene (low expression is related to relatively poor prognosis) and 2: oncogene-like gene (high expression is related to relatively poor prognosis.

Supplementary Table S5: Univariate and multivariate survival analyses of SWS-derived 22g-TAG signature providing the low- and high- grade IDC classification. Univariate and multivariate survival analyses of 22g-TAG signature and common clinical parameters using Cox proportional hazards model in four independent cohorts.

Supplementary Table S6: Gene ontology enrichment analysis of 22g-TAG molecular signature genes. Functionally enriched biological process, cellular components and pathways associated with the 22g-TAG genes using DAVID gene ontology tool.

Supplementary Table S7: Characteristics of genes and transcribed loci (represented by probesets) that are differentially expressed between HG1-like and HG3-like tumors, defined based on the 22g-TAG classifier. The fold changes represent the ratio of the median expression level in HG3-like with respect to the median of expression level in HG1-like tumors. A two-tailed Wilcoxon test was used to assess the significance of the difference of the gene expression profile between HG1-like and HG3-like tumors. Multiple probesets and transcribed isoforms can be associated with one gene.

Supplementary Table S8: Gene ontology and functional enrichment analysis for differentially expressed genes between HG1-like and HG3-like tumors. Gene ontology and functional enrichment analysis for up and down regulated genes in HG3-like tumors with respect to HG1-like tumors using DAVID Gene Ontology tool.

Supplementary Table S9: A list of differentially altered genes between HG1-like and HG3-like tumors. A list of 1,214 differentially altered genes between HG1-like and HG3-like tumors. The copy number intensity values were log2 transformed (diploid=1).

Supplementary Table S10: Contingency tables of the agreement between 22g-TAG derived genetic grades and classes resulting from unsupervised hierarchical clustering

4 × 2 table	Cluster 1	Cluster 2	Total
**HG1**	24	8	32
**HG1-like**	80	21	101
**HG3-like**	7	71	78
**HG3**	25	190	215
**Total**	136	290	426

2 × 2 table	Cluster 1	Cluster 2	Total
**LGG**	104	29	133
**HGG**	32	261	293
**Total**	136	290	426

Cohen's Kappa = 0.67, *p*-value = 6.257e-043

Contingency tables show frequencies produced by cross-classifying genetic grades and hierarchical clustering. The hierarchical clustering performed on 4,933 genes that were differentially expressed between HG1-like and HG3-like tumors using the Euclidian distance and average linkage agglomerative method.

Supplementary Table S11: lists of genes that were differentially expressed between LGG and HGG tumors. List of 3,073 and 2,618 genes that were up- and down-regulated in HGG tumors relative to LGG tumors. Each gene could be represented by more than one probeset or one probeset could represent multiple genes.

Supplementary Table S12: Gene ontology and functional enrichment analysis. Gene ontology and functional enrichment analysis for up- and down-regulated genes in HGG tumors with respect to LGG tumors using DAVID Gene Ontology tool.

Supplementary Table S13: A list of differentially altered genes between LGG and HGG tumors. Summary of copy number variation (CNV) profiles of 1,858 genes and their CNV event percentages in LGG and HGG tumors. Two-tailed Wilcoxon test p-values assess the difference between CNV profiles of LGG and HGG tumors.

Supplementary Table S14: Test of the agreement between DNA and RNA based sub-classification of HG2 tumors

		mRNA gene expression based classification		
	HG2 samples	**GG2**	**HG1-like**	**HG3-like**
DNA copy number based classification	**GG2**	1	6	9
	**HG1-like**	0	67	26
	**HG3-like**	3	28	42

Cohen's Kappa correlation coefficient = 0.32
p-value = 0.000074
A contingency table shows frequencies produced by cross-classifying the HG2 samples based on copy number variations and gene expression classifications. The statistical significance of the agreement between both classifications was assessed using Cohen's Kappa correlation coefficient.

Supplementary Table S15: enrichment analysis of stem cells related genes among molecular grading related genes. A. The significant enrichment of genes associated with 21 embryonic stem cells, as obtained by SAGE, in the genes that were differentially expressed between HG1-like and HG3-like tumors, specifically within the genes that are up regulated in HG3-like tumors with respect to HG1-like tumors. The analysis was performed using the DAVID gene ontology tool. **B.** list of 106 genes that are commonly expressed in all 21 embryonic stem cell lines obtained by SAGE. (only 84 genes are represented in Agilent G4502A_07_3 platform.)

Supplementary Table S16: Test of the agreement between genetic grades and clusters associated with stem cell related genes

4 × 2 table	cluster 1	cluster 2	Total
**HG1**	26	6	32
**HG1-like**	71	30	101
**HG3-like**	20	58	78
**HG3**	24	191	215
**Total**	141	285	426

2 × 2 table	cluster 1	cluster 2	Total
**LGG**	97	36	133
**HGG**	44	249	293
**Total**	141	285	426

Cohen's Kappa Coefficient = 0.57, *p*-value =3.295E-31

A contingency table shows frequencies produced by cross-classifying of patients based on genetic grades and classes resulting from unsupervised hierarchical clustering of the 106 genes. These genes are commonly expressed in the 21 embryonic stem cell lines studied in the CGAP SAGE database. Hierarchical clustering was performed using Euclidian distance and average linkage agglomerative method. The statistical significance of the agreement between both classifications was assessed using Cohen's Kappa correlation coefficient.

Supplementary Table S17: Summary of clinical parameters of 22g-TAG breast cancer patients' cohort and PCR primers used in qPCR validation. A. Summary of clinical parameters of patients' cohort used in the qPCR based grading validation of 22g-TAG signature. **B.** PCR primers sequences associated with 22g-TAG genes.
